# Risk of cardiovascular diseases in cancer patients: A nationwide representative cohort study in Taiwan

**DOI:** 10.1186/s12885-022-10314-y

**Published:** 2022-11-21

**Authors:** Tzu-Lin Yeh, Min-Shu Hsu, Hsin-Yin Hsu, Ming-Chieh Tsai, Jing-Rong Jhuang, Chun-Ju Chiang, Wen-Chung Lee, Kuo-Liong Chien

**Affiliations:** 1 Department of Family Medicine, Hsinchu MacKay Memorial Hospital, Hsinchu, Taiwan; 2 Institute of Epidemiology and Preventive Medicine, College of Public Health, National Taiwan University, Taipei, Taiwan; 3 Department of Medical Research, MacKay Memorial Hospital, New Taipei, Taiwan; 4 Department of Family Medicine, Taipei MacKay Memorial Hospital, Taipei, Taiwan; 5 Department of Medicine, MacKay Medical College, New Taipei City, Taiwan; 6 Division of Endocrinology, Department of Internal Medicine, MacKay Memorial Hospital, Tamsui Branch, New Taipei City, Taiwan; 7 Taiwan Cancer Registry, Taipei, Taiwan; 8 Innovation and Policy Center for Population Health and Sustainable Environment, College of Public Health, National Taiwan University, Taipei, Taiwan; 9 Department of Internal Medicine, National Taiwan University Hospital, Room 517, No.17, Xu-Zhou Rd, Taipei, Taiwan 10055

**Keywords:** Cancer patients, Cardiovascular disease, Cohort study, Onco-cardiology, Cardio-oncology

## Abstract

**Background:**

The associations with cancer and cardiovascular diseases (CVD) had inconsistent results. The study aimed to investigate the risk of cardiovascular diseases (CVD) between populations with and without cancer.

**Methods:**

Patients with common cancers in Taiwan were enrolled in the study between 2007 and 2018 using the Taiwan Cancer Registry. We focused on colorectal cancer, women’s breast cancer, lung cancer, liver cancer, oral cancer, prostate cancer, and thyroid cancers. The study endpoint was fatal and non-fatal CVD, which was defined as ischemic heart disease and ischemic stroke according to the National Health Insurance Research Database. We compared the risk of CVD between patients with cancer and age- and sex-matched (1:1 ratio) participants who did not have cancer or CVD. Multivariable adjusted hazard ratios (HRs) and 95% confidence intervals (CIs) were obtained from Cox regression analysis. To evaluate the chronological trend, we estimated the HRs and 95% CI yearly since the diagnosis.

**Results:**

Among the 552,485 cancer patients (mean age, 60.6 years; women, 47.7%) during the median follow-up period of 4.1 years, 32,634 cases of fatal and non-fatal CVD were identified. Compared with that noted in the non-cancer population, the overall fully adjusted HR with 95% CI was 1.28 (1.25, 1.30) in the cancer population. The CVD risk was the highest in the first year, the adjusted HR with 95% CI was 2.31 (2.23, 2.40), and this risk decreased yearly.

**Conclusions:**

Patients with cancer had a significantly higher risk of fatal or non-fatal CVD. The risk was the highest in the first year since diagnosis and decreased yearly.

**Supplementary Information:**

The online version contains supplementary material available at 10.1186/s12885-022-10314-y.

## Background

Cardiovascular disease (CVD) is the leading cause of death worldwide. Approximately 85% of CVD cases are due to ischemic heart disease (IHD) and stroke [1]. The traditionally well-known risk factors of CVD are hypertension, diabetes mellitus, and dyslipidemia. Cancers have recently been discovered to be strongly associated with CVD [2]. After surviving cancer, the chances of a patient dying a cancer-specific death is less likely than that of dying a CVD-specific death [3]. The risk of CVD was reported to be inconsistent among cancers [4, 5]. However, previous retrospective cohort studies have shown conflicting results: patients with cancer had a higher [6] or lower [7] risk of CVD than patients without cancers, men had a higher [6] or similar [8] risk as that of women, younger cancer patients had a higher [9] or lower [6] risk of CVD than the elderly, and the risk of CVD decreased [10] or increased [11] gradually after the diagnosis of cancer.

Asia accounted for the highest number of newly diagnosed cases of cancer in 2020 in both the sexes for all cancers [12]; the most common types being breast cancer and lung cancer, followed by colorectal, prostate, and stomach cancers. However, due to the increased frequency of risk factors for cancer [such as betel nut chewing, human papillomavirus [13], hepatitis B virus, and hepatitis C virus [14]] and a more detailed classification system of cancer prevalence in Taiwan, the common cancers in Taiwan are not similar to those worldwide. Liver, oral, and thyroid cancers were the common cancers in Taiwan [15]. However, previous studies regarding cancer and CVD in Taiwan have reported the association of a single cancer, such as colorectal [16] or nasopharyngeal cancer [17], with stroke [8, 18], with inconsistent results [16, 19].

This study aimed to investigate the risk of CVD in patients with common cancers in Taiwan, compare the risk of CVD over time, and perform subgroup analysis across sex and age to explore a potential effect modification.

## Materials and methods

This was a nationwide cohort study conducted between 2007 and 2019 in Taiwan. The target cancers were the seven most common cancers in Taiwan [15]: colorectal cancer, women’s breast cancer, lung cancer, liver cancer, oral cancer, prostate cancer, and thyroid cancer. Henceforth, the term “overall cancer patients” is used to indicate the sum of all the patients with the abovementioned cancers. Cancer exposures were confirmed by the International Classification of Diseases (ICD) for Oncology codes-3 in the National Taiwan Cancer Registry (TCR) database, which covered more than 98.4% of cancers in Taiwan [20] (Table S1). Clinically diagnosed prostate cancer rarely occurs before the age of 40, thus we excluded prostate cancer patients aged younger than 40 years. Prostate cancer patients aged ≥40 years and other cancer patients aged ≥20 years in the 2007-2018 TCR database were eligible and included in this study. Patients with missing or duplicated data, carcinoma in situ, double cancers, or established CVD were excluded.

The primary composite outcome, CVD, was defined as fatal or non-fatal IHD or ischemic stroke. IHD was defined as hospitalization with an ICD code or intervention with a procedure code of revascularization percutaneous coronary or coronary artery bypass graft (Tables S1 and S2). Outcomes were ascertained by the National Health Insurance Research Database (NHIRD), which covers 99.9% of the Taiwanese population [21] and the Taiwan’s National Death Registry between 2004 and 2019.

For each cancer patient, an age- and sex-matched, cancer-free, and CVD-free individual was selected from the NHIRD at a 1:1 ratio. The same index date with the corresponding cancer patient was assigned. Details on covariates of age, sex, cancer stage, and grade were obtained from the TCR database. The socio-economic status of urbanization, occupation, income, medical information of comorbidities, medication, and numbers of medical service used were retrieved from the NHIRD (Table S3). Our exposure cancer patients were retrieved from the TCR database, which covers 98.4% of all cancer patients in Taiwan. The comparison group was selected and the outcome was ascertained from the NHIRD, which covers 99.9% the whole population. Thus, our study is a nationwide representative cohort study in Taiwan.

### Statistical analyses

Categorical variables are presented as numbers and percentages and were analyzed using the chi-square test. Continuous variables are presented as mean and standard deviation and were analyzed using the t-test. Incidence rate was calculated as the number of event divided by 1000 person-year at risk. Person-years for each participant was calculated from the index date of initial cancer diagnosis until the date of occurrence of a CVD event, death, or on December 31, 2019, whichever came first. The risk of CVD in patients with or without cancer over time was demonstrated by Kaplan–Meier survival curves and was compared using the log-rank test.

Multivariable adjusted hazard ratios (HRs) and 95% confidence intervals (CIs) were estimated using Cox proportional hazards regression analysis. Proportional hazards assumption was not violated through the visual inspection of the log (−log (survival)) versus log of survival time plot (Fig. S1). The following models were applied: adjusted for sex and age (model 1); further adjusted for urbanization, occupation, and income (model 2); further adjusted for hypertension, diabetes mellitus, dyslipidemia, atrial fibrillation, aspirin use, anti-platelet agent use, anti-coagulant agent use, and number of medical service used (model 3).

To evaluate the chronological trend, we estimated the HRs and 95% CI yearly since the diagnosis. Aging is the shared risk factor of cancer and CVD, we set the cutoff age at 65 years as most of the older adults were defined [22]. Potential effect modifiers, sex, and age (cutoff: 65 years of age) were determined according to literature review [6, 9]. Due to the difference in inclusion criteria of age or sex, prostate cancer was excluded in the subgroup analysis of age, and breast and prostate cancers were excluded in the subgroup analysis of sex. *P* for interaction was obtained through the likelihood ratio test between a fully adjusted model with the interaction term and a nested model without the interaction term. To observe the differences in the sex-specific effect estimates, all the analyses in this study were stratified by sex. To test the robustness of our results, we performed two sensitivity analyses. We reported the risk of CVD, IHD, and stroke separately. Considering the competing risk of non-CVD deaths, cause-specific hazards competing risk model was performed [23].

All statistical tests were two-tailed with a type I error of 0.05, and a *P* value of 0.05 was considered statistically significant. Analyses were performed using SAS software (version 9.4; TS Level 1 M7) and Stata version 16.1 (Stata Corporation, College Station, TX77845, USA).

## Results

A total of 552,485 cancer patients were included after excluding patients with duplicated data (*n* = 78,465), double cancer (*n* = 52,417), carcinoma in situ (*n* = 45,080), unreasonable or missing data (*n* = 169), men with breast cancer (*n* = 461), not in our eligible age group (*n* = 1380), and established CVD (*n =* 56,588). The most common cancer was colorectal cancer (*n =* 113,986), followed by women’s breast cancer (*n* = 111,273), lung cancer (*n* = 101,286), liver cancer (*n* = 96,080), oral cancer (*n* = 62,731), prostate cancer (*n* = 35,804), and thyroid cancer (*n* = 31,325). The flow diagram is shown in Fig. S2.

Table 1 summarize the baseline characteristics of the overall cancer patients and the matched non-cancer patients. Only four cancer patients did not match their pairs. The mean (standard deviation) age of the overall cancer patients was 60.6 (±13.9) years, and 47.7% were women. Generally, our participants were more likely to have a blue-collar occupation and were domiciled in urban areas. The overall cancer patients were more prone to have a stage IV cancer (29.1%), have a well-differentiated grading (45.5%), use more medical services (49.4%), have relatively more comorbidities, and have higher medication use. Table S4 shows the baseline characteristics of patients with each cancer and of their matched non-cancer individuals. Prostate cancer patients had the highest mean age of 71.9 years, followed by lung cancer patients (65.5 years) and colorectal cancer patients (64.0 years), while thyroid cancer patients had the lowest mean age of 48.0 years. Thyroid cancer patients had the highest proportion of women at 77.2%, and liver and oral cancer patients had the lowest proportion of women at 29.9 and 8.8%, respectively. Most thyroid cancer patients were at stage I (74.0%), and most lung cancer patients were at stage IV (59.5%). The degree of differentiation was not mandatory to report in the thyroid cancer; thus, 90% of the covariates were missing in the TCR database.

**Table 1:** Baseline characteristics of populations with and without cancer

	Without cancer (*n =* 552,485)	Overall cancer (*n =* 552,485)	*P*
Age (years), mean (SD)	60.6 (13.9)	60.6 (13.9)	1
Women, n (%)	263,486 (47.7)	263,486 (47.7)	1
Occupation, n (%)			< 0.001
White collar	112,010 (20.3)	100,852 (18.3)	
Blue collar	236,777 (42.9)	234,294 (42.4)	
Others or missing	203,698 (36.9)	217,339 (39.3)	
Income (NTD/month), mean (SD)	22,813.3 (18,864.6)	23,561.7 (18,143.8)	< 0.001
Urbanization, n (%)	36,4123 (65.9)	331,491 (60.0)	< 0.001
Cancer stage, n (%)			NA
1	NA	134,987 (26.0)	
2	NA	119,293 (23.0)	
3	NA	113,781 (21.9)	
4	NA	151,359 (29.1)	
Grade, n (%)			NA
Good	NA	251,653 (45.5)	
Poor	NA	89,384 (16.2)	
Others or missing	NA	211,448 (38.3)	
Number of medical service uses in a year			< 0.001
0-19	371,628 (67.3)	96,130 (17.4)	
20-39	120,927 (21.9)	183,558 (33.2)	
≥ 40	59,930 (10.8)	272,797 (49.4)	
Comorbidities, n (%)			
Hypertension	12,883 (2.3)	44,206 (8.0)	< 0.001
Diabetes mellitus	5752 (1.0)	10,906 (2.0)	< 0.001
Dyslipidemia	11,437 (2.1)	10,098 (1.8)	< 0.001
Atrial fibrillation	20,891 (3.8)	20,874 (3.8)	0.93
Medication, n (%)			
Anti-platelet agent	133,352 (24.1)	133,189 (24.1)	0.72
Anti-coagulant agent	30,609 (5.5)	98,599 (17.8)	< 0.001
Aspirin	170,784 (30.9)	165,714 (30.0)	< 0.001

*NA* Not applicable, *NTD* New Taiwan Dollar, *SD* Standard deviation

**Fig. 1:**
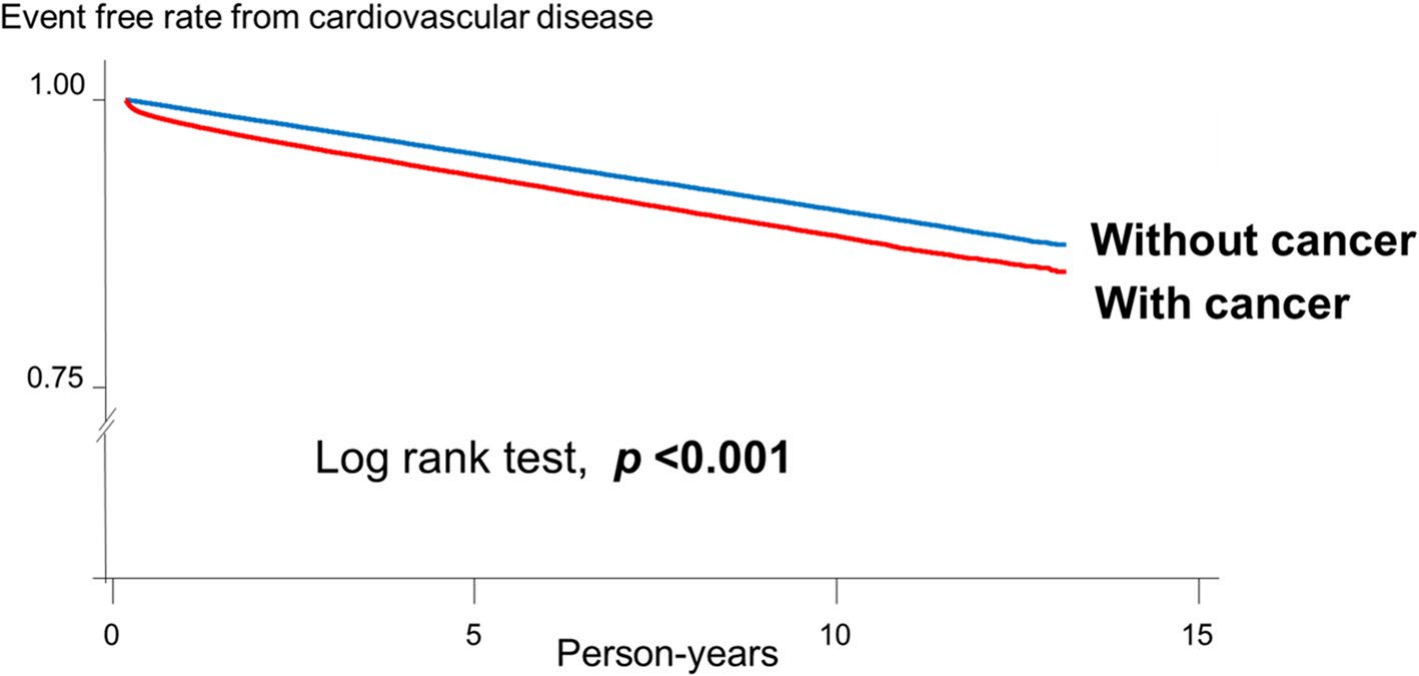
The Kaplan–Meier survival curves of cardiovascular disease. The Kaplan-Meier survival curves of cancer patients (red), and non-cancer population (blue) during the study (a maximum and median follow-up of 13 and 4.1 years, respectively)

We used the first event to calculate the person-years if a person had several events. The Kaplan–Meier survival curves of cancer are shown in Fig. 1. The CVD-free survival rate was significantly lower in cancer population than in the non-cancer population (log-rank test, *P* < 0.001). A total of 32,634 incident cases of fatal and non-fatal CVD in 2,154,515.8 person-years were documented in the overall cancer patients during a median (interquartile range) follow-up time of 4.1 (range 1.7–7.6) years. Table 2 shows that the incidence rate of CVD in the overall cancer patients was 15.1 per thousand person-years. All cancer patients had a significantly higher incidence rate of CVD than their non-cancer counterparts. Overall, compared with that noted in the sex- and age-matched non-cancer population, the cancer population had a fully adjusted HR with 95% CI of 1.28 (1.25, 1.30). Table S5 shows the incidence rate and Cox models of CVD in each cancer patient. The incidence rate of CVD was the highest in prostate and lung cancer patients (29.22 and 28.89 per 1000 person-years, respectively) and the lowest in breast and thyroid cancer patients (5.62 and 5.42 per 1000 person-years, respectively). Breast and thyroid cancer patients had a non-significant fully adjusted HR with 95% CI of 0.96 (0.90, 1.02) and 1.09 (0.97, 1.22), respectively.

**Table 2:** The risk of cardiovascular disease according to the presence of each target cancer

	Without cancer	Overall cancer
Participants, n	552,485	552,485
Fatal and non-fatal CVD, n	32,417	32,634
Person-years	3,185,885.6	2,154,515.8
Incident rate (per 1000 person-years)	10.2	15.1
Crude	1	1.43 (1.40, 1.45)
Model 1	1	1.59 (1.57, 1.62)
Model 2	1	1.56 (1.54, 1.59)
Model 3	1	1.28 (1.25, 1.30)

Model 1: Adjusted for age (20-24, 25-29, 30-34, 35-39, 40-44, 45-49, 50-54, 55-59, 60-64, 65-69, 70-74, 75-79, 80-84, ≥85 years) and sex

Model 2: Additional adjusted for occupation (white/blue collar), urbanization (yes/no), income (0-9999, 10,000-19,999, 20,000-29,999, 30,000-39,999, 40,000-49,999, ≥50,000 New Taiwan dollars in a month)

Model 3: Additional adjusted for hypertension, diabetes mellitus, hyperlipidemia, atrial fibrillation, aspirin use, anti-platelet agents use, anti-coagulant agents use, number of medical service uses

**Fig. 2:**
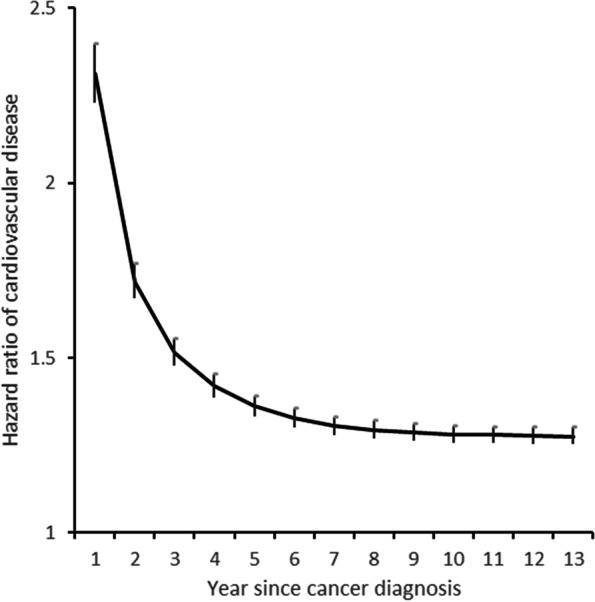
The risk of cardiovascular disease each year since cancer diagnosis. The hazard ratio (point) and 95% confidence interval (bar) of cardiovascular disease each year since cancer diagnosis

Figure 2 and Fig. S4 show the risk of CVD by year since cancer diagnosis. The detailed estimates and 95% CI of CVD are shown in Table S6. Overall, the risk of CVD was the highest in the first year since cancer diagnosis at 2.31 (2.23, 2.40) and decreased yearly. Lung cancer, liver and colorectal cancer had the highest risk of 4.28 (3.98, 4.61), 2.14 (1.97, 2.33) and 2.13 (1.98, 2.29), respectively, in the first year and showed a steeper slope. Prostate cancer patients had a relatively stable chronological trend of CVD.

**Fig. 3:**
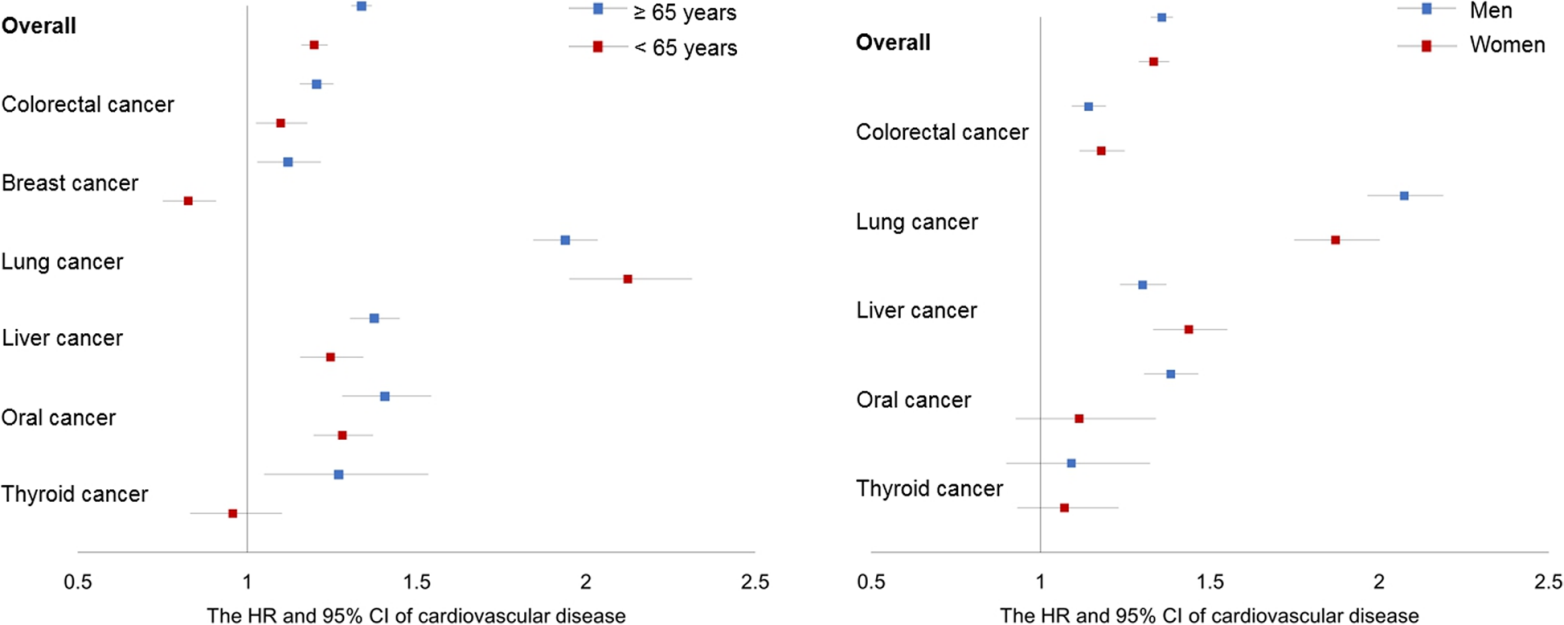
Subgroup analyses of the risk of cardiovascular disease. Fully-adjusted HRs (point) and 95% CIs (bars) for cardiovascular disease in overall and each cancer patients across age and sex groups. HR = hazard ratio; CI = confidence interval

Subgroup analysis (Table S7 and Fig. 3) revealed that the risk of CVD showed significant interactions with age. The fully adjusted HR with 95% CI was 1.20 (1.16, 1.24) in the non-elderly cancer patients, and 1.34 (1.31, 1.37) in the elderly cancer patients (*P* < .001 for interaction). Overall, the risk of CVD was not significantly different in terms of sex (*P* = .058 for interaction). The fully adjusted HR with 95% CI was 1.33 (1.29, 1.38) in women with cancer and 1.36 (1.33, 1.39) in men with cancer. The interaction effect of sex and age was different for each cancer. The results of all the analyses in the study stratified by sex are shown in Table S8-S14.

The risks of CVD, IHD, and stroke mortality and morbidity for the overall cancer patients are shown in Table S15. The risks of fatal and non-fatal IHD and stroke were still significantly higher in the cancer than in the non-cancer population. The fully adjusted HR with 95% CI were 1.20 (1.17, 1.23) and 1.27 (1.24, 1.30) for fatal and non-fatal IHD and stroke, respectively. Table S16 shows that considering the competing risk of non-CVD death, the fully adjusted HR with 95% CI was 1.32 (1.30, 1.35) in the overall cancer patients. The results of each cancer were similar to the main results of overall and each cancer.

## Discussion

Our study showed that patients with cancer had a significantly higher risk of CVD than their non-cancer counterparts. The risk of CVD was highest in lung cancer patients and was lowest in thyroid and breast cancer patients. The risk was the highest in the first year since cancer diagnosis, and this risk decreased yearly. Sex and age showed significant interactions with the risk of CVD with cancer. Based on the main findings, the risk of CVD was significantly higher in cancer patients, and this risk remained robust considering the competing risk.

Our findings were compatible with those of previous studies, in that cancer patients had a higher risk of stroke [4, 8, 9, 18, 24, 25] when affected by a single cancer or by multiple cancers. However, the risks of other CVD outcomes were not consistent, such that cancer patients had significantly higher [6, 26], lower [7, 27], or similar risks [10, 11, 28, 29] of heart diseases compared with that noted in the non-cancer population. CVD was defined as IHD and other heart diseases in one study [28] but as a composite endpoint of IHD, stroke, arrhythmia, venous thromboembolism, heart failure, cardiomyopathy, pericarditis, valvular heart disease, and peripheral vascular disease in another study [11]. Some studies focused on breast, colorectal, lung, pancreatic, or prostate cancers only [24], while other studies focused on 17 [4] or 20 [11] cancer types. The diverse definitions of CVD outcomes in various cancer types may explain the previously inconsistent results. Among different cancers, our findings were similar to those of previous studies, in that lung [5, 24] and liver [4] cancer patients had a relatively higher risk of CVD, while breast [28] and thyroid [11] cancer patients had a lower risk of CVD. Most studies revealed that the highest risk was at the time of cancer diagnosis and that then the risk decreased gradually [10, 30]. In a subgroup analysis of time since cancer diagnosis, the U shape was only observed in early-stage cancers but not in advanced-stage cancers [26]. An ascending risk of CVD was noted since a carcinoma in situ or stage I cancer diagnosis was made [24]. Most of our included cancer patients were at an advanced stage, as explained in our result. However, further studies are warranted to explore the interaction of cancer staging and time since diagnosis. Previous studies have rarely reported the interaction effect in subgroup analysis. One study showed that age and sex were not significant effect modifiers [28], indicating the need for further studies.

Age, obesity, smoking, and others were reported to be shared risk factors of cancer and CVD [31]. Cancer treatment-related cardiotoxicity, blood hyper-viscosity, conservative or suboptimal medical care, and the worse prognosis in a cancer patient [22] contribute to the cardio-oncology crossroad [32]. A U shape curve of stroke risk in the year after cancer diagnosis had been postulated [33]. The peak rose immediately due to cancer-mediated hypercoagulability, followed by a reduction in risk and then a progressive increase in risk due to the long-term effects of cancer treatments. In real-world settings, the curve interacted with cancer staging [26] and different CVD diseases [11]. Sex differences and aging also modify the association between CVD and cancer through visceral obesity [34], hyperinsulinemia, modifiable shared risk factors [35], and clonal hematopoiesis of indeterminate potential [36].

Our study provided evidence from real-world analysis to elucidate the risk of cancer-related CVD. The epidemiological evidence bridged basic and clinical research to improve patient care [37]. Clinically, a multidisciplinary cardiology and oncology health team can formulate clinical standard practice guidelines supported by epidemiological evidence. In basic research, a new anti-cancer drug could be developed with comprehensive cardiovascular safety and less cardiotoxicity for use in early diagnosis and prevention of CVD in cancer patients [38].

### Study Limitations

To the best of our knowledge, we report the first study of CVD risk in multiple cancers in Taiwan with a complete Cox model of years since cancer diagnosis and subgroup analyses. Our study has some limitations. First, residual confounders such as smoking, family history, and obesity existed. We made best efforts to collect the most relevant covariates in our database. Second, we focused only on the seven most common cancers. Further studies on more cancer types are warranted. Third, the level of evidence was limited in our retrospective cohort study. However, we ascertained these covariates and outcomes by medical records. Finally, considering the matched pairs in our methodology, frailty models should be used. However, due to large sample size and limited computing power of the hardware, we performed Cox model. The final results may be more conservative, but the basic trend toward protective or harmful will not change.

## Conclusion

In conclusion, our study demonstrated that cancer patients had a higher risk of CVD, especially lung cancer patients and in the first few years. Age was a significant effect modifier of overall cancer. Our study provided epidemiological evidence to elucidate the risk of cancer-related CVD, bridged basic and clinical research to improve patient care.

## Supplementary Information

**Additional file 1.**

## Data Availability

The datasets generated and/or analyzed during the current study are not publicly available due to the terms of consent to which the participants agreed but data are available from the authors upon reasonable request and with permission from the Health Promotion Administration at the Ministry of Health and Welfare in Taiwan.

## Abbreviations

CI: Confidence interval
CVD: Cardiovascular disease
HR: Hazard ratio
IHD: Ischemia heart disease
NHIRD: National Health Insurance Research Database
TCR: Taiwan Cancer Registry
