# Supplementary material for: Risk of cardiovascular diseases in cancer patients: A nationwide representative cohort study in Taiwan
Source: BMC Cancer. 2022 Nov 21;22:1198. doi: 10.1186/s12885-022-10314-y (PMC9677651; doi:10.1186/s12885-022-10314-y)
Supplement: Supplementary file 1 — Additional file 1. [file 12885_2022_10314_MOESM1_ESM.docx]

Supporting information

**Table S1.** **International Classification of Diseases codes used in the study cohort**

| **Exposure** | **International Classification of Diseases for Oncology codes-3** | |
| --- | --- | --- |
| Colorectal | C18-20 | |
| Breast | C50 | |
| Lung | C33-34 | |
| Liver | C22 | |
| Oral cavity | C00-06, C09-C10, C12-14 | |
| Prostate | C61 | |
| Thyroid | C73 | |
| **Outcome** | **ICD-9** | **ICD-10** |
| Carotid artery disease | 410, 411, 414.00, 414.01, 414.02, 414.03, 414.04, 414.05, v45.81, v45.82 | I21.01, I21.02, I21.09, I21.11, I21.19, I21.29, I21.21, I21.4, I21.3, I22.0, I22.1, I22.2, I22.8, I22.9, I20.0, I24.0, I24.1, I24.8, I24.9, I25.10, I25.750, I25.751, I25.758, I25.759, I25. 811, I25.10, I25.110, I25.111, I25.118, I25.119, I25.750, I25.751, I25.758, I25.759, I25.760, I25.761, I25.768, I25.769, I25. 811, I25.710, I25.711, I25.718, I25.719, I25.812, I25.731, I25.738, I25.739, 25.720, I25.721, I25.728, I25.729, I25.700, I25.701, I25.708, I25.709, I25.730**,** I25.760, I25.761, I25.768, I25.769, I25.790, I25.791, I25.798, I25.799, I25.810, I25.812, Z95.1, Z95.5, Z98.61 |
| Ischemia stroke | 433, 434, 435, 436, 437.1, 437.8, 437.9 | I66.01, I66.02, I66.03, I66.09, I66.11, I66.12, I66.13, I66.19, I66.21, I66.22, I66.23, I66.29, I66.3, I63.30, I63.311, I63.312, I63.319, I63.321, I63.322, I63.329, I63.331, I63.332, I63.339, I63.341, I63.342, I63.349, I63.39, I63.6, I66.9, I66.40, I66.411, I66.412, I66.419, I66.421, I66.422, I66.429, I66.431, I66.432, I66.439, I66.441, I66.442, I66.449, I66.49, I66.8, I66.9, I63.50, I63.511, I63.512, I63.519, I63.521, I63.522, I63.529, I63.531, I63.532, I63.539, I63.541, I63.542, I63.549, I63.59, I63.8, I63.9 I67.841, I67.848, I67.89, I67.81, I67.82, I67.9, G45.0, G45.8, G45.1, G45.2, G46.0, G46.1, G46.2, G45.9 |
| **Covariate** | **ICD-9** | **ICD-10** |
| Diabetes mellitus | 250 | E08, E09, E10, E11, E12, E13, E14 |
| Dyslipidemia | 272.1, 272.2, 272.3, 272.4 | E78.0, E78.1, E78.2, E78.3, E78.4, E78.5 |
| Hypertension | 401-405, 437.2 | I10, I11, I12, I13, I15, I16, I67.4 |
| Atrial fibrillation | 427.31 | I48.0, I48.1, I48.2, I48.9 |

ICD-9: International Classification of Diseases-9^th^ revision; ICD-10: International Classification of Diseases-10^th^ revision

**Table S2. Anatomical therapeutic chemical or procedure codes used in the study cohort**

| Outcome | Procedure codes |
| --- | --- |
| Revascularization percutaneous coronary intervention | 33076A, 33077A, 33078A, 33076B, 33077B, 33078B |
| Coronary artery bypass graft | 68023A, 68024A, 68025A, 68023B, 68024B, 68025B, N26002, N26003 |

**Table S3. Definitions of the covariates in the study cohort**

| Covariates | Definition in the Taiwan National Cancer Registry | Category |
| --- | --- | --- |
| Sex |  | Women/Men |
| Age | (Years old) | 20-24, 25-29, 30-34, 35-39, 40-44, 45-49, 50-54, 55-59, 60-64, 65-69, 70-74, 75-79, 80-84, ≥85 |
| Stage | If pathological staging was available, using the pathological staging; if not, using the clinical stating. Missing data were omitted. | 1, 2, 3, 4 |
| Grade | 1= Well or moderately differentiated;  2= Poorly differentiated or undifferentiated  3= Unknown or could not be differentiated or missing | 1, 2, 3 |
| Covariates | Definition in National Health Insurance Research Database | Category |
| Urbanization | Domicile in six urban area in Taiwan or not | Yes/No |
| Occupation | White collar: Officials in government agencies, public and private schools, employees of public or private enterprises or institutions, employee with a certain employer, employers or self-employed, professional or technical occupations  Blue collar: Those without a certain employer or self-employment, crew or captain, farmers, fishermen, soldiers, prisoner, low-income households  Others: occupation other than the above or missing data | White/Blue collar/Others |
| Income | (New Taiwan dollars in a month). Missing data were omitted. | 0-9,999, 10,000-19,999, 20,000-29,999, 30,000-39,999, 40,000-49,999, ≥50,000 |
| Numbers of medical uses | Total numbers of medical visit within one year of the index date, including outpatient clinic, hospitalization and emergent department medical visit | 0-19, 20-39, ≥40 |
| Hypertension | The use of following anti-hypertensive agents for at least 7 days continuously within one year of the index date: beta blocker, α2-agonist, α-blocker, sodium nitroprusside, diuretics, selective aldosterone receptor antagonist, calcium channel blocker, angiotensin-converting enzyme inhibitors, angiotensin receptor blocker, fixed-dose combinations, others (Minoxidil)) | Yes/No |
| Diabetes mellitus | The use of following anti-diabetic agents for at least 7 days continuously) within one year of the index date: Insulin, biguanide, sulfonylurea, α-glucosidase inhibitor, thiazolidinedione, glucagon-like peptide 1, sodium glucose co-transporters 2-inhibitor, dipeptidyl peptidase-4 inhibitors, fixed-dose combinations, others (Guar gum, Repaglinide, Nateglinide, Mitiglinide, calcium hydrate)) | Yes/No |
| Dyslipidemia | The use of following lipid-lowering agents for at least 7 days continuously) within one year of the index date: Statins, fibrate, cholestyramine resin, Probucol, Ezetimibe, proprotein convertase subtilisin/kexin type 9 inhibitors, fixed-dose combinations, others (Cholexamin, niacin)) | Yes/No |
| Atrial fibrillation | Either one discharge or twice outpatient clinic diagnosis of ICD codes | Yes/No |
| Aspirin | The use of aspirin for at least 7 days continuously | Yes/No |
| Anti-platelet agents | The use of the following for medications for at least 7 days continuously: Clopidogrel, Ticlopidine, Dipyridamole | Yes/No |
| Anti-coagulant agents | The use of the following for medications for at least 7 days continuously: Warfarin, Heparin, Dalteparin, Enoxaparin, Tinzaparin, Dabigatran, Rivaroxaban, Apixaban, Edoxaban | Yes/No |

# Table S4. Baseline characteristics of each cancer patients and the matched non-cancer population

|  | Without Colorectal  (n= 113,986) | Colorectal cancer (n= 113,986) | Without Breast  (n= 111,273) | Breast cancer (n= 111,273) | Without Lung  (n= 101,286) | Lung cancer (n= 101,286) | Without Liver cancer (n= 96,080) | Liver  (n= 96,080) | Without Oral  (n= 62,731) | Oral cancer (n= 62,731) | Without Prostate  (n= 35,804) | Prostate cancer (n= 35,804) | Without Thyroid  (n= 31,325) | Thyroid cancer (n= 31,325) |
| --- | --- | --- | --- | --- | --- | --- | --- | --- | --- | --- | --- | --- | --- | --- |
| Age (years) | 64.0 (13.7) | 64.0 (13.7) | 53.7 (11.8) | 53.7 (11.8) | 65.5 (12.6) | 65.5 (12.6) | 63.2 (12.5) | 63.2 (12.5) | 54.7 (11.5) | 54.7 (11.5) | 71.9 (9.2) | 71.9 (9.2) | 48.0 (13.4) | 48.0 (13.4) |
| Women | 63,713 (55.9) | 63,713 (55.9) | 111,273 (100.0) | 111,273 (100.0) | 43,520 (43.0) | 43,520 (43.0) | 28,724 (29.9) | 28,724 (29.9) | 5,518 (8.8) | 5,518 (8.8) | 0 (0.0) | 0 (0.0) | 24,178 (77.2) | 24,178 (77.2) |
| Occupation |  |  |  |  |  |  |  |  |  |  |  |  |  |  |
| White collar | 23,862 (20.9) | 21,522 (18.9) | 20,064 (18.0) | 17,864 (16.1) | 21,749 (21.5) | 19,288 (19.0) | 19,731 (20.5) | 16,386 (17.1) | 12,475 (19.9) | 14,490 (23.1) | 8,211 (22.9) | 7,113 (19.9) | 5,918 (18.9) | 4,189 (13.4) |
| Blue collar | 46,075 (40.4) | 47,920 (42.0) | 52,487 (47.2) | 58,721 (52.8) | 39,547 (39.0) | 39,356 (38.9) | 39,446 (41.1) | 34,374 (35.8) | 30,767 (49.0) | 21,267 (33.9) | 12,787 (35.7) | 14,924 (41.7) | 15,668 (50.0) | 17,732 (56.6) |
| Others or missing | 44,049 (38.6) | 44,544 (39.1) | 38,722 (34.8) | 34,688 (31.2) | 39,990 (39.5) | 42,642 (42.1) | 36,903 (38.4) | 45,320 (47.2) | 19,489 (31.1) | 26,974 (43.0) | 14,806 (41.4) | 13,767 (38.5) | 9,739 (31.1) | 9,404 (30.0) |
| Income (NTD/month) | 22,415.0 (19,015.6) | 23,767.9 (18,667.2) | 22,415.9 (16,914.4) | 24,695.3 (18,130.4) | 22,012.6 (18,864.1) | 23,323.5 (18,358.0) | 23,344.0 (19,657.1) | 22,837.5 (16,920.7) | 25,280.2 (20,392.1) | 19,605.3 (14,927.2) | 21,903.3 (19,947.1) | 26,515.0 (21,992.5) | 22,734.6 (17,438.5) | 26,321.3 (18,657.9) |
| Urbanization | 73,803 (64.7) | 70,182(61.6) | 76,951 (69.2) | 74,667(67.1) | 65,348 (64.5) | 60,076(59.3) | 62,458 (65.0) | 50,618(52.7) | 42,062 (67.1) | 32,369(51.6) | 22,057 (61.6) | 23,294(65.1) | 21,444 (68.5) | 20,285(64.8) |
| Stage |  |  |  |  |  |  |  |  |  |  |  |  |  |  |
| 1 | NA | 21,820 (19.6) | NA | 40,246 (36.4) | NA | 18,659 (18.7) | NA | 30,543 (32.6) | NA | 15,307 (24.8) | NA | 2,597 (7.4) | NA | 5,815 (74.0) |
| 2 | NA | 25,983 (23.4) | NA | 43,458 (39.3) | NA | 3,958 (4.0) | NA | 22,127 (23.6) | NA | 10,168 (16.5) | NA | 13,165 (37.8) | NA | 434 (5.5) |
| 3 | NA | 36,188 (32.5) | NA | 18,668 (16.9) | NA | 17,737 (17.8) | NA | 25,869 (27.6) | NA | 6,960 (11.3) | NA | 7,239 (20.8) | NA | 1,120 (14.3) |
| 4 | NA | 27,252 (24.5) | NA | 8,118 (7.3) | NA | 59,264 (**59.5**) | NA | 15,115 (16.1) | NA | 29,247 (**47.4**) | NA | 118,73 (34.0) | NA | 490 (6.2) |
| Grade |  |  |  |  |  |  |  |  |  |  |  |  |  |  |
| Good | NA | 81,272 (71.3) | NA | 61,499 (55.3) | NA | 26,691 (26.4) | NA | 23,769 (24.7) | NA | 42,401 (67.6) | NA | 13,495 (37.7) | NA | 2,526 (8.1) |
| Poor | NA | 9,268 (8.1) | NA | 28,511 (25.6) | NA | 17,582 (17.4) | NA | 10,476 (10.9) | NA | 6,104 (9.7) | NA | 16,849 (47.1) | NA | 594 (1.9) |
| Others or missing | NA | 23,446 (20.6) | NA | 21,263 (19.1) | NA | 57,013 (56.3) | NA | 61,835 (64.4) | NA | 142,26 (22.7) | NA | 5,460 (15.2) | NA | 28,205 (90.0) |
| Medical uses a year |  |  |  |  |  |  |  |  |  |  |  |  |  |  |
| 0-19 | 74016 (64.9) | 20,898 (18.3) | 77,069 (69.3) | 4,821 (4.3) | 64,208 (63.4) | 23,025 (22.7) | 63,262 (65.8) | 27,025 (28.1) | 47,485 (75.7) | 11,519 (18.4) | 21,868 (61.1) | 2,913 (8.1) | 23,720 (75.7) | 5,929 (18.9) |
| 20-39 | 26137 (22.9) | 42,792 (37.5) | 24,285 (21.8) | 30,075 (27.0) | 23,974 (23.7) | 27,747 (27.4) | 21,569 (22.4) | 34,473 (35.9) | 10,785 (17.2) | 19,135 (30.5) | 8,552 (23.9) | 12,975 (36.2) | 5,625 (18.0) | 16,361 (52.2) |
| ≥40 | 13833 (12.1) | 50,296 (44.1) | 9,919 (8.9) | 76,377 (68.6) | 13,104 (12.9) | 50,514 (49.9) | 11,249 (11.7) | 34,582 (36.0) | 4,461 (7.1) | 32,077 (51.1) | 5,384 (15.0) | 19,916 (55.6) | 1,980 (6.3) | 9,035 (28.8) |
| Comorbidities |  |  |  |  |  |  |  |  |  |  |  |  |  |  |
| Hypertension | 2626 (2.3) | 6,169 (5.4) | 2,592 (2.3) | 7,903 (7.1) | 2,303 (2.3) | 10,118 (10.0) | 2,337 (2.4) | 11,760 (12.2) | 1,615 (2.6) | 5,110 (8.1) | 757 (2.1) | 1,655 (4.6) | 653 (2.1) | 1,491 (4.8) |
| Diabetes mellitus | 1221 (1.1) | 1,885 (1.7) | 912 (0.8) | 2,006 (1.8) | 1,163 (1.1) | 2,214 (2.2) | 1,120 (1.2) | 1,874 (2.0) | 655 (1.0) | 1,934 (3.1) | 455 (1.3) | 629 (1.8) | 226 (0.7) | 364 (1.2) |
| Dyslipidemia | 2360 (2.1) | 2,503 (2.2) | 2,136 (1.9) | 2,114 (1.9) | 2,187 (2.2) | 1,327 (1.3) | 2,217 (2.3) | 1,193 (1.2) | 1,312 (2.1) | 1,221 (1.9) | 713 (2.0) | 945 (2.6) | 512 (1.6) | 795 (2.5) |
| Atrial fibrillation | 5275 (4.6) | 5,535 (4.9) | 2,005 (1.8) | 2,042 (1.8) | 4,872 (4.8) | 4,849 (4.8) | 4,434 (4.6) | 3,686 (3.8) | 1,699 (2.7) | 1,534 (2.4) | 2,186 (6.1) | 2,638 (7.4) | 420 (1.3) | 590 (1.9) |
| Medication |  |  |  |  |  |  |  |  |  |  |  |  |  |  |
| Anti-platelet | 31005 (27.2) | 30,917 (27.1) | 18,852 (16.9) | 17,320 (15.6) | 28,720 (28.4) | 26,877 (26.5) | 26,994 (24.3) | 27,518 (24.7) | 12,077 (19.3) | 12,104 (19.3) | 11,717 (32.7) | 14,023 (39.2) | 3,987 (12.7) | 4,430 (14.1) |
| Anticoagulant | 7405 (6.5) | 24,266 (21.3) | 3,457 (3.1) | 29,838 (26.8) | 6,794 (6.7) | 20,209 (20.0) | 6,407 (6.7) | 8,216 (8.6) | 2,815 (4.5) | 11,098 (17.7) | 2,991 (8.4) | 3,871 (10.8) | 740 (2.4) | 1,101 (3.5) |
| Aspirin | 39010 (34.2) | 38,327 (33.6) | 24,066 (21.6) | 22,352 (20.1) | 35,931 (35.5) | 34,056 (33.6) | 34,647 (36.1) | 30,386 (31.6) | 17,046 (27.2) | 16,231 (25.9) | 14,678 (41.0) | 18,175 (50.8) | 5,406 (17.3) | 6,187 (19.8) |

Categorical variables were presented with numbers and percentage, continuous variables were presented with mean and standard deviation. NA, not applicable; New Taiwan Dollar

# Table S5. The risk of cardiovascular disease according to the presence of each target cancer.

|  | Without Colorectal cancer | Colorectal cancer | Without Breast cancer | Breast cancer | Without Lung cancer | Lung cancer | Without Liver cancer | Liver cancer | Without Oral cancer | Oral cancer | Without Prostate cancer | Prostate cancer | Without Thyroid cancer | Thyroid cancer |
| --- | --- | --- | --- | --- | --- | --- | --- | --- | --- | --- | --- | --- | --- | --- |
| Participants, n | 113,986 | 113,986 | 111,273 | 111,273 | 101,286 | 101,286 | 96,080 | 96,080 | 62,731 | 62,731 | 35,804 | 35,804 | 31,325 | 31,325 |
| Fatal and non-fatal CVD, n | 7,637 | 8,417 | 3,273 | 3,448 | 7,133 | 6,360 | 7,241 | 5,022 | 3,534 | 3,963 | 2,843 | 4,453 | 756 | 971 |
| Person-years | 640,450 | 474,042 | 662,249 | 613,718 | 562,702 | 220,122 | 581,580 | 254,113 | 389,391 | 260,834 | 167,324 | 152,380 | 182,191 | 179,307 |
| Incident rate (per 1,000 person-years) | 11.92 | 17.76 | 4.94 | 5.62 | 12.68 | 28.89 | 12.45 | 19.76 | 9.08 | 15.19 | 16.99 | 29.22 | 4.15 | 5.42 |
| Crude | 1 | **1.44 (1.40, 1.48)** | 1 | **1.14 (1.09, 1.19)** | 1 | **1.96 (1.89, 2.03)** | 1 | **1.50 (1.44, 1.56)** | 1 | **1.65 (1.57, 1.72)** | 1 | **1.70 (1.62, 1.78)** | 1 | **1.30 (1.19, 1.43)** |
| Model 1 | 1 | **1.50 (1.45, 1.54)** | 1 | **1.15 (1.09, 1.20)** | 1 | **2.23 (2.15, 2.31)** | 1 | **1.54 (1.49, 1.60)** | 1 | **1.70 (1.62, 1.78)** | 1 | **1.72 (1.64, 1.80)** | 1 | **1.38 (1.25, 1.52)** |
| Model 2 | 1 | **1.46 (1.41, 1.51)** | 1 | **1.15 (1.10, 1.21)** | 1 | **2.18 (2.10, 2.26)** | 1 | **1.51 (1.45, 1.56)** | 1 | **1.66 (1.59, 1.74)** | 1 | **1.67 (1.60, 1.75)** | 1 | **1.39 (1.26, 1.53)** |
| Model 3 | 1 | **1.15 (1.11, 1.19)** | 1 | 0.96 (0.90, 1.02) | 1 | **1.98 (1.90, 2.07)** | 1 | **1.34 (1.29, 1.40)** | 1 | **1.35 (1.28, 1.43)** | 1 | **1.08 (1.02, 1.14)** | 1 | 1.09 (0.97, 1.22) |

Bold font indicates a significant risk; presented with hazard ratio with 95% confidence interval

Model 1: Adjusted for age (20-24, 25-29, 30-34, 35-39, 40-44, 45-49, 50-54, 55-59, 60-64, 65-69, 70-74, 75-79, 80-84, ≥85 years old) and sex

Model 2: Additional adjusted for occupation (white/blue collar), urbanization (yes/no), income (0-9,999, 10,000-19,999, 20,000-29,999, 30,000-39,999, 40,000-49,999, ≥50,000 New Taiwan dollars in a month)

Model 3: Additional adjusted for hypertension, diabetes mellitus, hyperlipidemia, atrial fibrillation, aspirin use, anti-platelet agents use, anti-coagulant agents use, number of medical uses

**Table S6. The risk of cardiovascular disease each year since cancer diagnosis**

| Year since cancer diagnosis | Without cancer | Overall cancer | Without Colorectal cancer | Colorectal cancer | Without Breast cancer | Breast cancer | Without Lung cancer | Lung cancer | Without Liver cancer | Liver cancer | Without Oral cancer | Oral cancer | Without Prostate cancer | Prostate cancer | Without Thyroid cancer | Thyroid cancer |
| --- | --- | --- | --- | --- | --- | --- | --- | --- | --- | --- | --- | --- | --- | --- | --- | --- |
| Year 1 | 1 | **2.31 (2.23, 2.40)** | 1 | **2.13 (1.98, 2.29)** | 1 | **1.43 (1.25, 1.64)** | 1 | **4.28 (3.98, 4.61)** | 1 | **2.14 (1.97, 2.33)** | 1 | **2.06 (1.82, 2.32)** | 1 | **1.20 (1.08, 1.34)** | 1 | **1.59 (1.24, 2.04)** |
| Year 2 | 1 | **1.72 (1.67, 1.77)** | 1 | **1.55 (1.46, 1.63)** | 1 | **1.14 (1.02, 1.26)** | 1 | **3.06 (2.89, 3.25)** | 1 | **1.67 (1.56, 1.78)** | 1 | **1.63 (1.49, 1.79)** | 1 | **1.14 (1.05, 1.25)** | 1 | **1.20 (1.00, 1.45)** |
| Year 3 | 1 | **1.52 (1.48, 1.55)** | 1 | **1.37 (1.31, 1.44)** | 1 | 1.03 (0.94, 1.12) | 1 | **2.58 (2.45, 2.72)** | 1 | **1.51 (1.42, 1.60)** | 1 | **1.44 (1.33, 1.56)** | 1 | **1.14 (1.06, 1.22)** | 1 | **1.15 (0.98, 1.35)** |
| Year 4 | 1 | **1.42 (1.39, 1.45)** | 1 | **1.28 (1.22, 1.33)** | 1 | 1.01 (0.93, 1.09) | 1 | **2.35 (2.24, 2.47)** | 1 | **1.43 (1.36, 1.51)** | 1 | **1.39 (1.30, 1.50)** | 1 | **1.12 (1.05, 1.20)** | 1 | **1.12 (0.97, 1.30)** |
| Year 5 | 1 | **1.36 (1.33, 1.39)** | 1 | **1.21 (1.17, 1.27)** | 1 | 0.98 (0.91, 1.06) | 1 | **2.20 (2.10, 2.31)** | 1 | **1.40 (1.34, 1.47)** | 1 | **1.38 (1.29, 1.48)** | 1 | **1.10 (1.03, 1.17)** | 1 | **1.11 (0.97, 1.27)** |
| Year 6 | 1 | **1.33 (1.30, 1.36)** | 1 | **1.19 (1.15, 1.24)** | 1 | 0.94 (0.88, 1.01) | 1 | **2.12 (2.03, 2.22)** | 1 | **1.37 (1.31, 1.44)** | 1 | **1.35 (1.27, 1.44)** | 1 | **1.11 (1.05, 1.18)** | 1 | **1.11 (0.97, 1.26)** |
| Year 7 | 1 | **1.31 (1.28, 1.33)** | 1 | **1.19 (1.14, 1.23)** | 1 | 0.94 (0.88, 1.01) | 1 | **2.06 (1.97, 2.16)** | 1 | **1.35 (1.29, 1.42)** | 1 | **1.35 (1.27, 1.43)** | 1 | **1.09 (1.03, 1.16)** | 1 | **1.07 (0.95, 1.21)** |
| Year 8 | 1 | **1.30 (1.27, 1.32)** | 1 | **1.18 (1.14, 1.22)** | 1 | 0.94 (0.88, 1.00) | 1 | **2.03 (1.95, 2.12)** | 1 | **1.35 (1.29, 1.41)** | 1 | **1.35 (1.27, 1.43)** | 1 | **1.09 (1.03, 1.15)** | 1 | **1.07 (0.95, 1.21)** |
| Year 9 | 1 | **1.29 (1.26, 1.31)** | 1 | **1.17 (1.13, 1.21)** | 1 | 0.94 (0.89, 1.01) | 1 | **2.00 (1.92, 2.09)** | 1 | **1.34 (1.29, 1.40)** | 1 | **1.36 (1.29, 1.44)** | 1 | **1.08 (1.02, 1.14)** | 1 | **1.07 (0.95, 1.20)** |
| Year 10 | 1 | **1.28 (1.26, 1.30)** | 1 | **1.16 (1.12, 1.21)** | 1 | 0.95 (0.90, 1.02) | 1 | **1.99 (1.91, 2.08)** | 1 | **1.34 (1.29, 1.40)** | 1 | **1.35 (1.28, 1.43)** | 1 | **1.08 (1.02, 1.14)** | 1 | **1.08 (0.97, 1.22)** |
| Year 11 | 1 | **1.28 (1.26, 1.30)** | 1 | **1.16 (1.12, 1.20)** | 1 | 0.96 (0.90, 1.02) | 1 | **1.99 (1.91, 2.07)** | 1 | **1.35 (1.29, 1.40)** | 1 | **1.36 (1.28, 1.44)** | 1 | **1.08 (1.02, 1.14)** | 1 | **1.09 (0.97, 1.22)** |
| Year 12 | 1 | **1.28 (1.25, 1.30)** | 1 | **1.15 (1.11, 1.19)** | 1 | 0.96 (0.90, 1.02) | 1 | **1.99 (1.90, 2.07)** | 1 | **1.34 (1.29, 1.40)** | 1 | **1.36 (1.29, 1.44)** | 1 | **1.08 (1.02, 1.14)** | 1 | **1.10 (0.98, 1.23)** |
| Year 13 | 1 | **1.28 (1.25, 1.30)** | 1 | **1.15 (1.11, 1.19)** | 1 | 0.96 (0.90, 1.02) | 1 | **1.98 (1.90, 2.07)** | 1 | **1.34 (1.29, 1.40)** | 1 | **1.35 (1.28, 1.43)** | 1 | **1.08 (1.02, 1.14)** | 1 | **1.09 (0.97, 1.22)** |

Bold font indicates a significant risk; presented with hazard ratio with 95% confidence interval

Adjusted by model 3: Adjusted for age, sex, occupation, urbanization, income, hypertension, diabetes mellitus, hyperlipidemia, atrial fibrillation, aspirin use, anti-platelet agents use, anti-coagulant agents use, number of medical uses

# Table S7. Subgroup analyses of the risk of cardiovascular disease according to the presence of cancer

|  | Without cancer | Overall cancer | Without breast cancer | Breast cancer | Without oral cancer | Oral cancer | Without colorectal cancer | Colorectal cancer | Without Lung cancer | Lung  cancer | Without Liver cancer | Liver cancer | Without Thyroid cancer | Thyroid cancer |
| --- | --- | --- | --- | --- | --- | --- | --- | --- | --- | --- | --- | --- | --- | --- |
| Sex | *P* = 0.58 for interaction | | NA | | *P* = 0.015 for interaction | | *P* = 0.009 for interaction | | *P* = 0.49 for interaction | | *P* = 0.006 for interaction | | *P* = 1 for interaction | |
| Women | 1 | **1.33 (1.29, 1.38)** |  |  | 1 | 1.11 (0.93, 1.34) | 1 | **1.18 (1.12, 1.25)** | 1 | **1.87 (1.75, 2.00)** | 1 | **1.44 (1.33, 1.55)** | 1 | 1.07 (0.93, 1.23) |
| Men | 1 | **1.36 (1.33, 1.39)** |  |  | 1 | **1.38 (1.31, 1.47)** | 1 | **1.14 (1.09, 1.19)** | 1 | **2.07 (1.97, 2.19)** | 1 | **1.30 (1.24, 1.37)** | 1 | 1.09 (0.90, 1.32) |
| Age | *P* < 0.001 for interaction | | *P* = 0.033 for interaction | | *P* = 0.28 for interaction | | *P* = 0.13for interaction | | *P* < 0.001 for interaction | | *P* = 0.13 for interaction | | *P* = 0.32 for interaction | |
| <65 | 1 | **1.20 (1.16, 1.24)** | 1 | **0.83 (0.75, 0.91**) | 1 | **1.28 (1.20, 1.37)** | 1 | **1.10 (1.03, 1.18)** | 1 | **2.12 (1.95, 2.31)** | 1 | **1.25 (1.16, 1.34)** | 1 | 0.96 (0.83, 1.10) |
| ≥65 | 1 | **1.34 (1.31, 1.37)** | 1 | **1.12 (1.03, 1.22)** | 1 | **1.41 (1.28, 1.54)** | **1** | **1.20 (1.16, 1.25)** | 1 | **1.94 (1.85, 2.04)** | 1 | **1.38 (1.30, 1.45)** | 1 | **1.27 (1.05, 1.54)** |

**Bold** font indicates a significant risk; presented with hazard ratio with 95% confidence interval

Adjusted by model 3: Adjusted for age, sex, occupation, urbanization, income, hypertension, diabetes mellitus, hyperlipidemia, atrial fibrillation, aspirin use, anti-platelet agents use, anti-coagulant agents use, number of medical uses

# Table S8. Baseline characteristics of populations with and without cancer in each sex

|  | Women | | | Men | | |
| --- | --- | --- | --- | --- | --- | --- |
|  | Without cancer  (n=263,486) | With cancer  (n=263,486) | *P* | Without cancer  (n=288,999) | With cancer  (n=288,999) | *P* |
| Age (years), mean (SD) | 58.6 (14.1) | 58.6 (14.1) | 1 | 62.5 (13.4) | 62.5 (13.4) | 1 |
| Occupation, n (%) |  |  | <0.001 |  |  | <0.001 |
| White collar | 49,248 (18.7) | 42,895 (16.3) |  | 62,762 (21.7) | 57,957 (20.1) |  |
| Blue collar | 113,864 (43.2) | 121,843 (46.2) |  | 122,913 (42.5) | 112,451 (38.9) |  |
| Others or missing | 100,374 (38.1) | 98,748 (37.5) |  | 103,324 (35.8) | 118,591 (41.4) |  |
| Income (NTD/month), mean (SD) | 21,902.5 (17,032.2) | 23,753.3 (17,528.7) | <0.001 | 23,643.6 (20,356.8) | 23,387.0 (18,685.2) | <0.001 |
| Urbanization, n (%) | 178,023(67.6) | 167,108(63.4) | <0.001 | 186,100(64.4) | 164,383(56.9) | <0.001 |
| Cancer stage, n (%) |  |  | NA |  |  | NA |
| 1 | NA | 77,930 (32.2) |  | NA | 57,057 (20.6) |  |
| 2 | NA | 64,300 (26.6) |  | NA | 54,993 (19.8) |  |
| 3 | NA | 47,580 (19.7) |  | NA | 66,201 (23.8) |  |
| 4 | NA | 51,932 (21.5) |  | NA | 99,427 (35.8) |  |
| Grade, n (%) |  |  | NA |  |  | NA |
| Good | NA | 124,128 (47.1) |  | NA | 127,525 (44.1) |  |
| Poor | NA | 42,098 (16.0) |  | NA | 47,286 (16.4) |  |
| Others or missing | NA | 97,260 (36.9) |  | NA | 114,188 (39.5) |  |
| Number of medical service uses in a year |  |  | <0.001 |  |  | <0.001 |
| 0-19 | 170,652 (64.8) | 33,626 (12.8) |  | 200,976 (69.5) | 62,504 (21.6) |  |
| 20-39 | 63,116 (24.0) | 86,467 (32.8) |  | 57,811 (20.0) | 97,091 (33.6) |  |
| ≥40 | 29,718 (11.3) | 143,393 (54.4) |  | 30,212 (10.5) | 129,404 (44.8) |  |
| Comorbidities, n (%) |  |  |  |  |  |  |
| Hypertension | 5,846 (2.2) | 17,606 (6.7) | <0.001 | 7,037 (2.4) | 26,600 (9.2) | <0.001 |
| Diabetes mellitus | 2,511 (1.0) | 4,254 (1.6) | <0.001 | 3,241 (1.1) | 6,652 (2.3) | <0.001 |
| Dyslipidemia | 5,396 (2.1) | 4,677 (1.8) | <0.001 | 6,041 (2.1) | 5,421 (1.9) | <0.001 |
| Atrial fibrillation | 8,078 (3.1) | 7,677 (2.9) | 0.0012 | 12,813 (4.4) | 13,197 (4.6) | 0.0148 |
| Medication, n (%) |  |  |  |  |  |  |
| Anti-platelet agent | 58,938 (24.1) | 57,907 (24.1) | 0.0006 | 74,414 (25.8) | 75,282 (26.1) | 0.0092 |
| Anti-coagulant agent | 12,014 (4.6) | 53,308 (20.2) | <0.001 | 18,595 (6.4) | 45,291 (15.7) | <0.001 |
| Aspirin | 72,459 (27.5) | 69,626 (26.4) | <0.001 | 98,325 (34.0) | 96,088 (33.3) | <0.001 |

NA= not applicable; NTD= New Taiwan Dollar; SD= standard deviation.

# Table S9. The risk of cardiovascular disease according to the presence of each target cancer in each sex

|  | Women | | Men | |
| --- | --- | --- | --- | --- |
|  | Without cancer | Without cancer | Overall cancer | Overall cancer |
| Participants, n | 263,486 | 263,486 | 288,999 | 288,999 |
| Fatal and non-fatal CVD, n | 11,374 | 11,538 | 21,043 | 21,096 |
| Person-years | 1,538,282.4 | 1,191,719.6 | 1,647,603.2 | 962,796.3 |
| Incident rate (per 1,000 person-years) | 7.39 | 9.68 | 12.77 | 21.91 |
| Crude | 1 | **1.28 (1.24, 1.31)** | 1 | **1.63 (1.59, 1.66)** |
| Model 1 | 1 | **1.48 (1.44, 1.51)** | 1 | **1.68 (1.65, 1.71)** |
| Model 2 | 1 | **1.45 (1.41, 1.49)** | 1 | **1.65 (1.62, 1.68)** |
| Model 3 | 1 | **1.23 (1.19, 1.26)** | 1 | **1.31 (1.28, 1.34)** |

Bold font indicates a significant risk; presented with hazard ratio with 95% confidence interval

Model 1: Adjusted for age (20-24, 25-29, 30-34, 35-39, 40-44, 45-49, 50-54, 55-59, 60-64, 65-69, 70-74, 75-79, 80-84, ≥85 years) and sex

Model 2: Additional adjusted for occupation (white/blue collar), urbanization (yes/no), income (0-9,999, 10,000-19,999, 20,000-29,999, 30,000-39,999, 40,000-49,999, ≥50,000 New Taiwan dollars in a month)

Model 3: Additional adjusted for hypertension, diabetes mellitus, hyperlipidemia, atrial fibrillation, aspirin use, anti-platelet agents use, anti-coagulant agents use, number of medical service uses

# Table S10. The risk of cardiovascular disease according to the presence of each target cancer in each sex

|  | Women | | Men | | Women | | Men | | Women | | Men | | Women | | Men | | Women | | Men | |
| --- | --- | --- | --- | --- | --- | --- | --- | --- | --- | --- | --- | --- | --- | --- | --- | --- | --- | --- | --- | --- |
|  | Without Colorectal cancer | Colorectal cancer | Without Colorectal cancer | Colorectal cancer | Without Lung cancer | Lung cancer | Without Lung cancer | Lung cancer | Without Liver cancer | Liver cancer | Without Liver cancer | Liver cancer | Without Oral cancer | Oral cancer | Without Oral cancer | Oral cancer | Without Thyroid cancer | Thyroid cancer | Without Thyroid cancer | Thyroid cancer |
| Participants, n | 50,273 | 50,273 | 63,713 | 63,713 | 43,520 | 43,520 | 57,766 | 57,766 | 28,724 | 28,724 | 67,356 | 67,356 | 5,518 | 5,518 | 57,213 | 57,213 | 24,178 | 24,178 | 7,147 | 7,147 |
| Fatal and non-fatal CVD, n | 2,812 | 3,083 | 4,825 | 5,334 | 2,450 | 2,526 | 4,683 | 3,834 | 2,083 | 1,583 | 5,158 | 3,439 | 270 | 289 | 3,264 | 3,674 | 486 | 609 | 270 | 362 |
| Person-years | 289,087 | 216,003 | 351,363 | 258,039 | 239,558 | 117,185 | 323,144 | 102,937 | 172,279 | 79,000 | 409,300 | 175,112 | 32,444 | 24,871 | 356,947 | 235,964 | 142,666 | 140,942 | 39,525 | 38,364 |
| Incident rate | 9.73 | 14.27 | 13.73 | 20.67 | 10.23 | 21.56 | 14.49 | 37.25 | 12.09 | 20.04 | 12.60 | 19.64 | 8.32 | 11.62 | 9.14 | 15.57 | 3.41 | 4.32 | 6.83 | 9.44 |
| Crude | 1 | **1.42 (1.35, 1.49)** | 1 | **1.45 (1.40, 1.51)** | 1 | **1.90 (1.80, 2.02)** | 1 | **2.13 (2.04, 2.23)** | 1 | **1.56 (1.46, 1.67)** | 1 | **1.47 (1.41, 1.54)** | 1 | **1.36 (1.15, 1.61)** | 1 | **1.68 (1.60, 1.76)** | 1 | **1.27 (1.13, 1.43)** | 1 | **1.38 (1.18, 1.61)** |
| Model 1 | 1 | **1.48 (1.40, 1.56)** | 1 | **1.50 (1.44, 1.56)** | 1 | **2.13 (2.01, 2.26)** | 1 | **2.29 (2.19, 2.40)** | 1 | **1.69 (1.58, 1.81)** | 1 | **1.49 (1.42, 1.56)** | 1 | **1.46 (1.23, 1.72)** | 1 | **1.72 (1.64, 1.80)** | 1 | **1.33 (1.18, 1.50)** | 1 | **1.45 (1.23, 1.69)** |
| Model 2 | 1 | **1.43 (1.36, 1.51)** | 1 | **1.47 (1.42, 1.53)** | 1 | **2.09 (1.97, 2.22)** | 1 | **2.25 (2.14, 2.35)** | 1 | **1.64 (1.53, 1.75)** | 1 | **1.46 (1.40, 1.53)** | 1 | **1.38 (1.17, 1.64)** | 1 | **1.69 (1.61, 1.78)** | 1 | **1.34 (1.19, 1.51)** | 1 | **1.46 (1.25, 1.72)** |
| Model 3 | 1 | **1.18 (1.12, 1.25)** | 1 | **1.14 (1.09, 1.19)** | 1 | **1.87 (1.75, 2.00)** | 1 | **2.07 (1.96, 2.19)** | 1 | **1.44 (1.33, 1.55)** | 1 | **1.30 (1.24, 1.37)** | 1 | 1.11 (0.93, 1.34) | 1 | **1.38 (1.31, 1.47)** | 1 | 1.07 (0.93, 1.23) | 1 | 1.09 (0.90, 1.32) |

Incident rate of per 1,000 person-years, bold font indicates a significant risk; presented with hazard ratio with 95% confidence interval. Model 1: Adjusted for age (20-24, 25-29, 30-34, 35-39, 40-44, 45-49, 50-54, 55-59, 60-64, 65-69, 70-74, 75-79, 80-84, ≥85 years) and sex.Model 2: Additional adjusted for occupation (white/blue collar), urbanization (yes/no), income (0-9,999, 10,000-19,999, 20,000-29,999, 30,000-39,999, 40,000-49,999, ≥50,000 New Taiwan dollars in a month). odel 3: Additional adjusted for hypertension, diabetes mellitus, hyperlipidemia, atrial fibrillation, aspirin use, anti-platelet agents use, anti-coagulant agents use, number of medical service uses

# Table S11. The risk of cardiovascular disease each year since cancer diagnosis in each sex

|  | Women | | Men | | Women | | Men | | Women | | Men | | Women | | Men | | Women | | Men | | Women | | Men | |
| --- | --- | --- | --- | --- | --- | --- | --- | --- | --- | --- | --- | --- | --- | --- | --- | --- | --- | --- | --- | --- | --- | --- | --- | --- |
| Year since cancer diagnosis | Without cancer | Overall cancer | Without cancer | Overall cancer | Without Colorectal cancer | Colorectal cancer | Without Colorectal cancer | Colorectal cancer | Without Lung cancer | Lung cancer | Without Lung cancer | Lung cancer | Without Liver cancer | Liver cancer | Without Liver cancer | Liver cancer | Without Oral cancer | Oral cancer | Without Oral cancer | Oral cancer | Without Thyroid cancer | Thyroid cancer | Without Thyroid cancer | Thyroid cancer |
| Year 1 | 1 | **2.41 (2.27, 2.57)** | 1 | **2.25 (2.15, 2.35)** | 1 | **2.32 (2.06, 2.61)** | 1 | **2.03 (1.86, 2.23)** | 1 | **4.04 (3.56, 4.57)** | 1 | **4.46 (4.07, 4.89)** | 1 | **2.43 (2.10, 2.81)** | 1 | **2.00 (1.81, 2.21)** | 1 | **2.10 (1.42, 3.10)** | 1 | **2.07 (1.82, 2.35)** | 1 | **1.88 (1.37, 2.59)** | 1 | 1.14 (0.76, 1.71) |
| Year 2 | 1 | **1.72 (1.64, 1.81)** | 1 | **1.71 (1.65, 1.77)** | 1 | **1.63 (1.49, 1.78)** | 1 | **1.50 (1.40, 1.61)** | 1 | **2.86 (2.59, 3.15)** | 1 | **3.21 (2.98, 3.45)** | 1 | **1.84 (1.64, 2.06)** | 1 | **1.58 (1.46, 1.71)** | 1 | **1.46 (1.09, 1.97)** | 1 | **1.66 (1.51, 1.83)** | 1 | **1.35 (1.06, 1.71)** | 1 | 0.97 (0.71, 1.31) |
| Year 3 | 1 | **1.51 (1.44, 1.57)** | 1 | **1.52 (1.47, 1.57)** | 1 | **1.42 (1.31, 1.54)** | 1 | **1.36 (1.28, 1.44)** | 1 | **2.43 (2.23, 2.65)** | 1 | **2.69 (2.52, 2.87)** | 1 | **1.68 (1.52, 1.86)** | 1 | **1.43 (1.33, 1.53)** | 1 | **1.33 (1.02, 1.73)** | 1 | **1.46 (1.34, 1.59)** | 1 | **1.25 (1.02, 1.54)** | 1 | 0.97 (0.74, 1.27) |
| Year 4 | 1 | **1.40 (1.35, 1.45)** | 1 | **1.43 (1.39, 1.47)** | 1 | **1.31 (1.22, 1.41)** | 1 | **1.26 (1.19, 1.33)** | 1 | **2.27 (2.09, 2.46)** | 1 | **2.42 (2.27, 2.57)** | 1 | **1.55 (1.42, 1.70)** | 1 | **1.38 (1.29, 1.47)** | 1 | 1.23 (0.97, 1.56) | 1 | **1.41 (1.31, 1.53)** | 1 | 1.17 (0.97, 1.40) | 1 | 1.01 (0.79, 1.29) |
| Year 5 | 1 | **1.33 (1.29, 1.38)** | 1 | **1.38 (1.34, 1.41)** | 1 | **1.25 (1.17, 1.34)** | 1 | **1.20 (1.14, 1.26)** | 1 | **2.08 (1.93, 2.24)** | 1 | **2.30 (2.16, 2.44)** | 1 | **1.53 (1.40, 1.67)** | 1 | **1.34 (1.27, 1.43)** | 1 | 1.19 (0.96, 1.48) | 1 | **1.41 (1.31, 1.51)** | 1 | 1.18 (0.99, 1.39) | 1 | 0.98 (0.78, 1.23) |
| Year 6 | 1 | **1.28 (1.24, 1.33)** | 1 | **1.35 (1.32, 1.39)** | 1 | **1.22 (1.15, 1.30)** | 1 | **1.18 (1.12, 1.24)** | 1 | **2.01 (1.87, 2.17)** | 1 | **2.21 (2.09, 2.34)** | 1 | **1.49 (1.37, 1.62)** | 1 | **1.31 (1.24, 1.39)** | 1 | 1.10 (0.90, 1.35) | 1 | **1.39 (1.30, 1.48)** | 1 | 1.13 (0.97, 1.33) | 1 | 1.03 (0.83, 1.27) |
| Year 7 | 1 | **1.26 (1.22, 1.31)** | 1 | **1.33 (1.30, 1.36)** | 1 | **1.22 (1.15, 1.30)** | 1 | **1.17 (1.12, 1.23)** | 1 | **1.96 (1.83, 2.11)** | 1 | **2.14 (2.03, 2.27)** | 1 | **1.46 (1.35, 1.59)** | 1 | **1.30 (1.23, 1.38)** | 1 | 1.10 (0.90, 1.33) | 1 | **1.38 (1.30, 1.47)** | 1 | 1.10 (0.94, 1.28) | 1 | 1.00 (0.81, 1.23) |
| Year 8 | 1 | **1.24 (1.20, 1.28)** | 1 | **1.33 (1.30, 1.36)** | 1 | **1.20 (1.13, 1.27)** | 1 | **1.18 (1.12, 1.23)** | 1 | **1.92 (1.79, 2.06)** | 1 | **2.12 (2.01, 2.24)** | 1 | **1.45 (1.34, 1.56)** | 1 | **1.30 (1.23, 1.38)** | 1 | 1.11 (0.92, 1.35) | 1 | **1.38 (1.30, 1.47)** | 1 | 1.08 (0.93, 1.25) | 1 | 1.03 (0.84, 1.26) |
| Year 9 | 1 | **1.24 (1.20, 1.28)** | 1 | **1.32 (1.29, 1.35)** | 1 | **1.20 (1.13, 1.27)** | 1 | **1.16 (1.11, 1.21)** | 1 | **1.89 (1.76, 2.02)** | 1 | **2.10 (1.99, 2.21)** | 1 | **1.44 (1.33, 1.55)** | 1 | **1.30 (1.23, 1.37)** | 1 | 1.14 (0.94, 1.38) | 1 | **1.39 (1.31, 1.48)** | 1 | 1.06 (0.92, 1.23) | 1 | 1.04 (0.85, 1.27) |
| Year 10 | 1 | **1.23 (1.19, 1.27)** | 1 | **1.31 (1.28, 1.34)** | 1 | **1.19 (1.13, 1.26)** | 1 | **1.15 (1.10, 1.20)** | 1 | **1.87 (1.75, 2.01)** | 1 | **2.09 (1.98, 2.21)** | 1 | **1.44 (1.33, 1.55)** | 1 | **1.30 (1.24, 1.37)** | 1 | 1.13 (0.94, 1.36) | 1 | **1.38 (1.30, 1.46)** | 1 | 1.07 (0.93, 1.24) | 1 | 1.07 (0.88, 1.31) |
| Year 11 | 1 | **1.23 (1.19, 1.27)** | 1 | **1.31 (1.28, 1.34)** | 1 | **1.18 (1.12, 1.25)** | 1 | **1.15 (1.10, 1.20)** | 1 | **1.87 (1.74, 2.00)** | 1 | **2.08 (1.98, 2.20)** | 1 | **1.44 (1.34, 1.56)** | 1 | **1.30 (1.24, 1.37)** | 1 | 1.13 (0.94, 1.37) | 1 | **1.39 (1.31, 1.47)** | 1 | 1.07 (0.93, 1.23) | 1 | 1.08 (0.89, 1.31) |
| Year 12 | 1 | **1.23 (1.19, 1.27)** | 1 | **1.31 (1.28, 1.34)** | 1 | **1.18 (1.12, 1.25)** | 1 | **1.14 (1.09, 1.19)** | 1 | **1.87 (1.75, 2.00)** | 1 | **2.08 (1.97, 2.19)** | 1 | **1.44 (1.33, 1.55)** | 1 | **1.30 (1.24, 1.37)** | 1 | 1.13 (0.94, 1.35) | 1 | **1.39 (1.31, 1.47)** | 1 | 1.08 (0.94, 1.25) | 1 | 1.09 (0.90, 1.32) |
| Year 13 | 1 | **1.23 (1.19, 1.26)** | 1 | **1.31 (1.28, 1.34)** | 1 | **1.18 (1.12, 1.25)** | 1 | **1.14 (1.09, 1.19)** | 1 | **1.87 (1.75, 2.00)** | 1 | **2.07 (1.96, 2.19)** | 1 | **1.44 (1.33, 1.55)** | 1 | **1.30 (1.24, 1.37)** | 1 | 1.11 (0.93, 1.34) | 1 | **1.38 (1.31, 1.47)** | 1 | 1.07 (0.93, 1.23) | 1 | 1.09 (0.90, 1.32) |

Bold font indicates a significant risk; presented with hazard ratio with 95% confidence interval

Adjusted by model 3: Adjusted for age, sex, occupation, urbanization, income, hypertension, diabetes mellitus, hyperlipidemia, atrial fibrillation, aspirin use, anti-platelet agents use, anti-coagulant agents use, number of medical uses

# Table S12. Further subgroup analyses of the risk of cardiovascular disease according to the presence of cancer

|  | Without cancer | Overall cancer | Without oral cancer | Oral cancer | Without colorectal cancer | Colorectal cancer | Without Lung cancer | Lung  cancer | Without Liver cancer | Liver cancer | Without Thyroid cancer | Thyroid cancer |
| --- | --- | --- | --- | --- | --- | --- | --- | --- | --- | --- | --- | --- |
| Total | **P < 0.001** for interaction | | P = 0.28 for interaction | | P = 0.13 for interaction | | **P < 0.001** for interaction | | P = 0.13 for interaction | | P = 0.32 for interaction | |
| Age <65 years | 1 | **1.20 (1.16, 1.24)** | 1 | **1.28 (1.20, 1.37)** | 1 | **1.10 (1.03, 1.18)** | 1 | **2.12 (1.95, 2.31)** | 1 | **1.25 (1.16, 1.34)** | 1 | 0.96 (0.83, 1.10) |
| Age ≥65 years | 1 | **1.34 (1.31, 1.37)** | 1 | **1.41 (1.28, 1.54)** | 1 | **1.20 (1.16, 1.25)** | 1 | **1.94 (1.85, 2.04)** | 1 | **1.38 (1.30, 1.45)** | 1 | **1.27 (1.05, 1.54)** |
| Women | **P = 0.004** for interaction | | P= 0.86 for interaction | | P = 0.46 for interaction | | **P < 0.001** for interaction | | P = 0.12 for interaction | | P = 1 for interaction | |
| Age <65 years | 1 | **1.27 (1.18, 1.36)** | 1 | 0.99 (0.71, 1.37) | 1 | **1.15 (1.01, 1.32)** | 1 | **2.10 (1.83, 2.42)** | 1 | **1.29 (1.08, 1.54)** | 1 | 1.00 (0.83, 1.19) |
| Age ≥65 years | 1 | **1.33 (1.28, 1.39)** | 1 | 1.22 (0.97, 1.53) | 1 | **1.22 (1.14, 1.29)** | 1 | **1.79 (1.65, 1.93)** | 1 | **1.43 (1.31, 1.55)** | 1 | 1.20 (0.96, 1.49) |
| Men | **P = 0.004** for interaction | | P= 0.10 for interaction | | P = 0.46 for interaction | | **P = 0.034** for interaction | | P = 0.085 for interaction | | P = 0.10 for interaction | |
| Age <65 years | 1 | **1.27 (1.22, 1.32)** | 1 | **1.30 (1.21, 1.40)** | 1 | **1.08 (1.00, 1.17)** | 1 | **2.15 (1.93, 2.40)** | 1 | **1.23 (1.13, 1.34)** | 1 | 0.91 (0.72, 1.14) |
| Age ≥65 years | 1 | **1.39 (1.35, 1.43)** | 1 | **1.47 (1.32, 1.62)** | 1 | **1.19 (1.13, 1.26)** | 1 | **2.05 (1.92, 2.18)** | 1 | **1.33 (1.24, 1.42)** | 1 | **1.48 (1.02, 2.14)** |

Bold font indicates a significant risk; presented with hazard ratio with 95% confidence interval

Adjusted by model 3: Adjusted for age, sex, occupation, urbanization, income, hypertension, diabetes mellitus, hyperlipidemia, atrial fibrillation, aspirin use, anti-platelet agents use, anti-coagulant agents use, number of medical uses

# Table S13. The risk of cardiovascular disease mortality and morbidity according to the presence of cancer in each sex

|  | Cardiovascular disease | | | | Ischemia heart disease | | | | Ischemia stroke | | | |
| --- | --- | --- | --- | --- | --- | --- | --- | --- | --- | --- | --- | --- |
|  | Women | | Men | | Women | | Men | | Women | | Men | |
|  | Without cancer | With cancer | Without cancer | With cancer | Without cancer | With cancer | Without cancer | With cancer | Without cancer | With cancer | Without cancer | With cancer |
| Participants | 263,486 | 263,486 | 288,999 | 288,999 | 270,431 | 270,431 | 302,781 | 302,781 | 272,459 | 272,459 | 310,078 | 310,078 |
| Fatal and non-fatal CVD | 11,374 | 11,538 | 21,043 | 21,096 | 7,278 | 7,016 | 14,961 | 14,445 | 6,537 | 6,060 | 11,396 | 10,042 |
| Person-years | 1,538,282.4 | 1,191,719.6 | 1,647,603.2 | 962,796.3 | 1,613,883.5 | 1,220,213.6 | 1,783,082.0 | 1,011,633.9 | 1,630,336.4 | 1,236,292.7 | 1,852,600.9 | 1,053,826.4 |
| Incident rate | 7.39 | 9.68 | 12.77 | 21.91 | 4.51 | 5.75 | 8.39 | 14.28 | 4.01 | 4.90 | 6.15 | 9.53 |
| Crude | 1 | **1.28 (1.24, 1.31)** | 1 | **1.63 (1.59, 1.66)** | 1 | **1.24 (1.20, 1.28)** | 1 | **1.60 (1.57, 1.64)** | 1 | **1.20 (1.16, 1.24)** | 1 | **1.49 (1.45, 1.53)** |
| Model 1 | 1 | **1.48 (1.44, 1.51)** | 1 | **1.68 (1.65, 1.71)** | 1 | **1.44 (1.40, 1.49)** | 1 | **1.67 (1.63, 1.70)** | 1 | **1.43 (1.38, 1.48)** | 1 | **1.56 (1.52, 1.61)** |
| Model 2 | 1 | **1.45 (1.41, 1.49)** | 1 | **1.65 (1.62, 1.68)** | 1 | **1.42 (1.38, 1.47)** | 1 | **1.64 (1.61, 1.68)** | 1 | **1.40 (1.36, 1.46)** | 1 | **1.53 (1.48, 1.57)** |
| Model 3 | 1 | **1.23 (1.19, 1.26)** | 1 | **1.31 (1.28, 1.34)** | 1 | **1.11 (1.07, 1.15)** | 1 | **1.25 (1.22, 1.29)** | 1 | **1.28 (1.23, 1.34)** | 1 | **1.26 (1.22, 1.30)** |

Incident rate of per 1,000 person-years. Bold font indicates a significant risk; presented with hazard ratio with 95% confidence interval

Model 1: Adjusted for age (20-24, 25-29, 30-34, 35-39, 40-44, 45-49, 50-54, 55-59, 60-64, 65-69, 70-74, 75-79, 80-84, ≥85 years old) and sex

Model 2: Additional adjusted for occupation (white/blue collar), urbanization (yes/no), income (0-9,999, 10,000-19,999, 20,000-29,999, 30,000-39,999, 40,000-49,999, ≥50,000 New Taiwan dollars in a month)

Model 3: Additional adjusted for hypertension, diabetes mellitus, hyperlipidemia, atrial fibrillation, aspirin use, anti-platelet agents use, anti-coagulant agents use, number of medical uses

# Table S14. The risk of cardiovascular disease according to the presence of cancer considering the competing risk in each sex

|  | Women | | Men | | Women | | Men | | Women | | Men | | Women | | Men | | Women | | Men | | Women | | Men | |
| --- | --- | --- | --- | --- | --- | --- | --- | --- | --- | --- | --- | --- | --- | --- | --- | --- | --- | --- | --- | --- | --- | --- | --- | --- |
|  | Without cancer | Overall cancer | Without cancer | Overall cancer | Without colorectal cancer | Colorectal cancer | Without colorectal cancer | Colorectal cancer | Without lung cancer | Lung cancer | Without lung cancer | Lung cancer | Without liver cancer | Liver cancer | Without liver cancer | Liver cancer | Without oral cancer | Oral cancer | Without oral cancer | Oral cancer | Without thyroid cancer | Thyroid cancer | Without thyroid cancer | Thyroid cancer |
| Cox | 1 | **1.23 (1.19, 1.26)** | 1 | **1.31 (1.28, 1.34)** | 1 | **1.18 (1.12, 1.25)** | 1 | **1.14 (1.09, 1.19)** | 1 | **1.87 (1.75, 2.00)** | 1 | **2.07 (1.96, 2.19)** | 1 | **1.44 (1.33, 1.55)** | 1 | **1.30 (1.24, 1.37)** | 1 | **1.38 (1.31, 1.47** | 1 | 1.11 (0.93, 1.34) | 1 | 1.07 (0.93, 1.23) | 1 | 1.09 (0.90, 1.32) |
| Cause-specific | 1 | **1.29 (1.25, 1.32)** | 1 | **1.34 (1.32, 1.37)** | 1 | **1.23 (1.16, 1.30)** | 1 | **1.20 (1.15, 1.25)** | 1 | **1.96 (1.83, 2.10)** | 1 | **2.17 (2.06, 2.29**) | 1 | **1.44 (1.33, 1.55)** | 1 | **1.30 (1.24, 1.37)** | 1 | 1.12 (0.94, 1.35) | 1 | **1.46 (1.38, 1.55**) | 1 | 1.08 (0.94, 1.24) | 1 | 1.11 (0.92, 1.35) |

Bold font indicates a significant risk; presented with hazard ratio with 95% confidence interval

Adjusted by model 3: Adjusted for age, sex, occupation, urbanization, income, hypertension, diabetes mellitus, hyperlipidemia, atrial fibrillation, aspirin use, anti-platelet agents use, anti-coagulant agents use, number of medical uses

# Table S15. The risk of cardiovascular disease mortality and morbidity according to the presence of cancer

|  | Cardiovascular disease | | Ischemia heart disease | | Ischemia stroke | |
| --- | --- | --- | --- | --- | --- | --- |
|  | Without cancer | With cancer | Without cancer | With cancer | Without cancer | With cancer |
| Participants | 552,485 | 552,485 | 573,212 | 573,212 | 582,537 | 582,537 |
| Fatal and non-fatal CVD | 32,417 | 32,634 | 22,239 | 21,461 | 17,933 | 16,102 |
| Person-years | 3,185,885.6 | 2,154,515.8 | 3,396,965.5 | 2,231,847.5 | 3,482,937.3 | 2,290,119.2 |
| Incident rate (per 1,000 person-years) | 10.2 | 15.1 | 6.55 | 9.62 | 5.15 | 7.03 |
| Crude | 1 | **1.43 (1.40, 1.45)** | 1 | **1.40 (1.37, 1.43)** | 1 | **1.33 (1.30, 1.35)** |
| Model 1 | 1 | **1.59 (1.57, 1.62)** | 1 | **1.58 (1.55, 1.61)** | 1 | **1.50 (1.47, 1.53)** |
| Model 2 | 1 | **1.56 (1.54, 1.59)** | 1 | **1.55 (1.52, 1.58)** | 1 | **1.47 (1.44, 1.50)** |
| Model 3 | 1 | **1.28 (1.25, 1.30)** | 1 | **1.20 (1.17, 1.23)** | 1 | **1.27 (1.24, 1.30)** |

Bold font indicates a significant risk; presented with hazard ratio with 95% confidence interval.

Model 1: Adjusted for age (20-24, 25-29, 30-34, 35-39, 40-44, 45-49, 50-54, 55-59, 60-64, 65-69, 70-74, 75-79, 80-84, ≥85 years old) and sex

Model 2: Additional adjusted for occupation (white/blue collar), urbanization (yes/no), income (0-9,999, 10,000-19,999, 20,000-29,999, 30,000-39,999, 40,000-49,999, ≥50,000 New Taiwan dollars in a month). Model 3: Additional adjusted for hypertension, diabetes mellitus, hyperlipidemia, atrial fibrillation, aspirin use, anti-platelet agents use, anti-coagulant agents use, number of medical uses

# Table S16. The risk of cardiovascular disease according to the presence of cancer considering the competing risk

|  | Without cancer | Overall cancer | Without colorectal cancer | Colorectal cancer | Without breast cancer | Breast cancer | Without lung cancer | Lung cancer | Without liver cancer | Liver cancer | Without oral cancer | Oral cancer | Without prostate cancer | Prostate cancer | Without thyroid cancer | Thyroid cancer |
| --- | --- | --- | --- | --- | --- | --- | --- | --- | --- | --- | --- | --- | --- | --- | --- | --- |
| Cox | 1 | **1.28 (1.25, 1.30)** | 1 | **1.15 (1.11, 1.19)** | 1 | 0.96 (0.90, 1.02) | 1 | **1.98 (1.90, 2.07)** | 1 | **1.34 (1.29, 1.40)** | 1 | **1.35 (1.28, 1.43)** | 1 | **1.08 (1.02, 1.14)** | 1 | 1.09 (0.97, 1.22) |
| Cause-specific | 1 | **1.32 (1.30, 1.35)** | 1 | **1.21 (1.17, 1.25)** | 1 | 1.06 (1.00, 1.13) | 1 | **2.08 (2.00, 2.17)** | 1 | **1.34 (1.29, 1.40)** | 1 | **1.43 (1.35, 1.51)** | 1 | **1.09 (1.03, 1.15**) | 1 | 1.10 (0.99, 1.23) |

Bold font indicates a significant risk; presented with hazard ratio with 95% confidence interval

Adjusted for age (20-24, 25-29, 30-34, 35-39, 40-44, 45-49, 50-54, 55-59, 60-64, 65-69, 70-74, 75-79, 80-84, ≥85 years old), sex, occupation (white/blue collar), urbanization (yes/no), income (0-9,999, 10,000-19,999, 20,000-29,999, 30,000-39,999, 40,000-49,999, ≥50,000 New Taiwan dollars in a month), hypertension, diabetes mellitus, hyperlipidemia, atrial fibrillation, aspirin use, anti-platelet agents use, anti-coagulant agents use, number of medical uses

**Figure S1.** **The logarithm negative logarithm plot against logarithm of time for proportional hazard assumption**

**
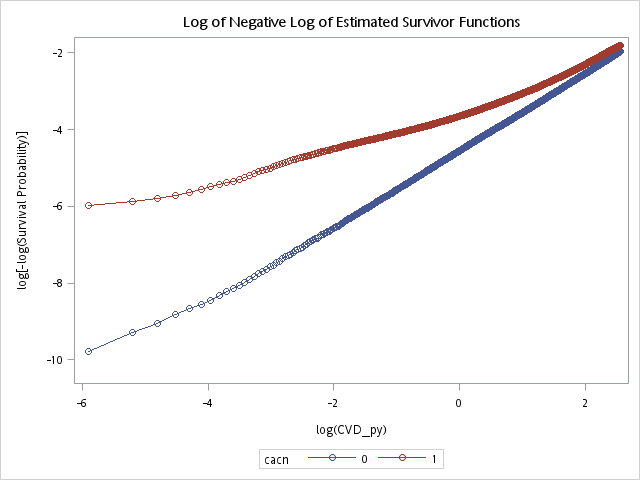
**

**Figure S2. Flow diagram of the participants enrollment**

**
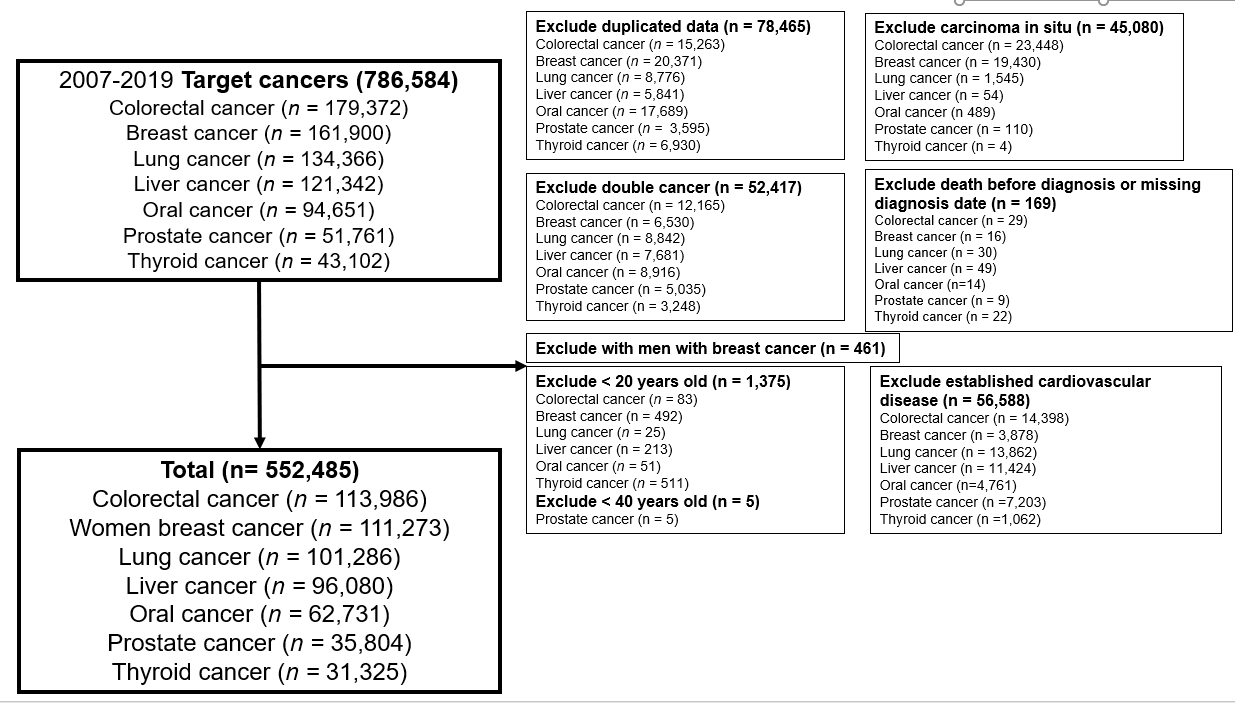
**

**Figure S3.** **The Kaplan-Meier survival curves of cardiovascular disease of each cancer**


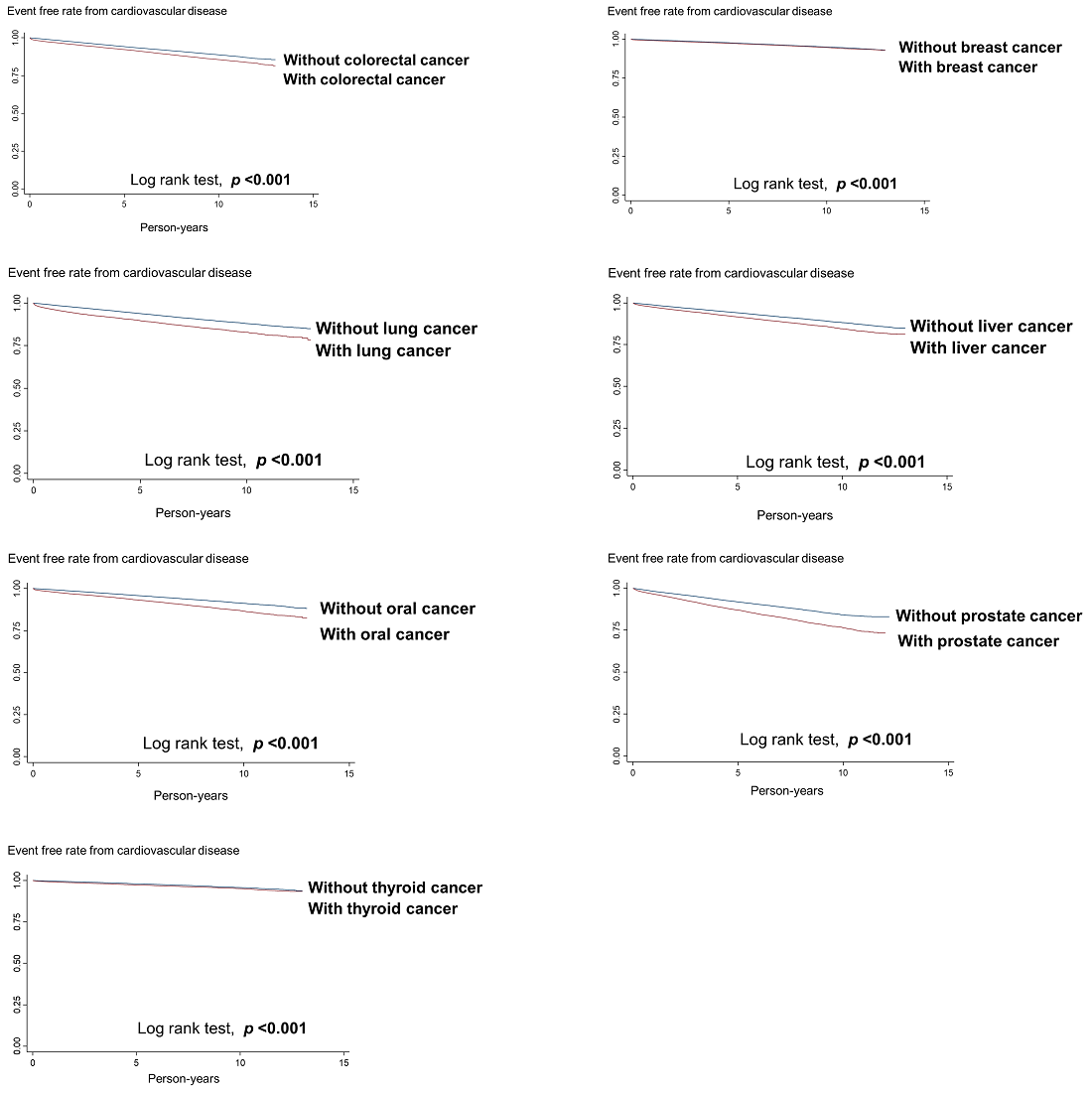


**Figure S4.** **The risk of cardiovascular disease each year since each cancer diagnosis**

**
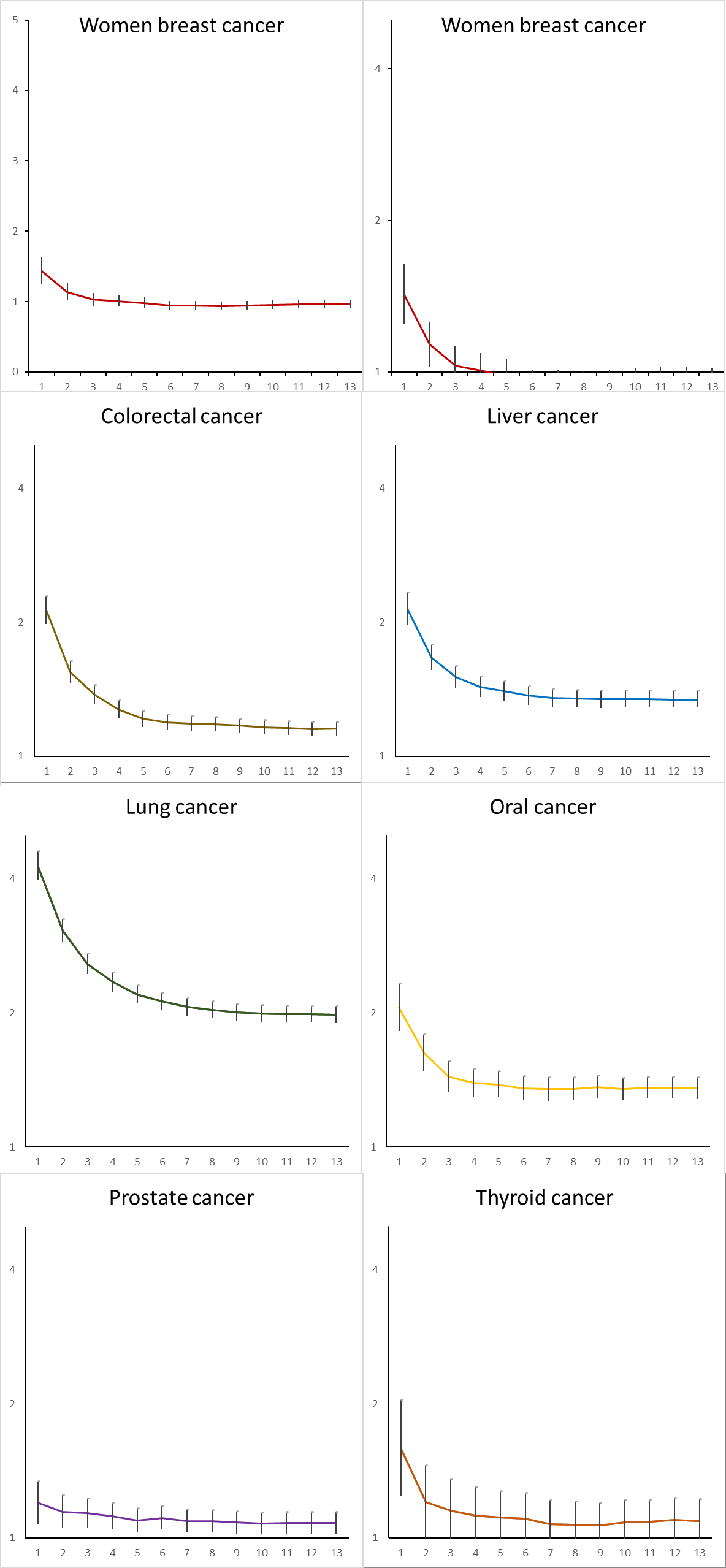
**

X axis: year since diagnosis; Y axis: hazard ratio of the risk of cardiovascular disease in logarithmic scale. The figure of breast cancer was presented in both logarithmic and general scale due to the hazard ratio <1
